# Early-life stress alters affective behaviors in adult mice through persistent activation of CRH-BDNF signaling in the oval bed nucleus of the stria terminalis

**DOI:** 10.1038/s41398-020-01070-3

**Published:** 2020-11-11

**Authors:** Pu Hu, Isabella Maita, Mimi L. Phan, Edward Gu, Christopher Kwok, Andrew Dieterich, Mark M. Gergues, Christine N. Yohn, Yu Wang, Jiang-Ning Zhou, Xin-Rui Qi, Dick F. Swaab, Zhiping P. Pang, Paul J. Lucassen, Troy A. Roepke, Benjamin A. Samuels

**Affiliations:** 1 Department of Psychology, Rutgers, The State University of New Jersey, Piscataway, NJ 08854 USA; 2 CAS Key Laboratory of Brain Function and Diseases, Life Science School, University of Science and Technology of China, Hefei, 230027 China; 3 Center for Translational Neurodegeneration and Regenerative Therapy, Shanghai Tenth People’s Hospital Affiliated to Tongji University School of Medicine, Shanghai, 200072 China; 4 Department of Neuropsychiatric Disorders, Netherlands Institute for Neuroscience, An Institute of the Royal Netherlands Academy of Arts and Sciences, Meibergdreef, Amsterdam 1105 BA The Netherlands; 5 Department of Neuroscience and Cell Biology, Rutgers Robert Wood Johnson Medical School, New Brunswick, NJ 08901 USA; 6 Brain Plasticity Group, Swammerdam Institute for Life Sciences, Center for Neuroscience, University of Amsterdam, 1098 XH Amsterdam, The Netherlands; 7 Department of Animal Sciences, School of Environmental and Biological Sciences, Rutgers, The State University of New Jersey, New Brunswick, NJ 08901 USA; 8 Present Address: Neuroscience Graduate Program, University of California, San Francisco, San Francisco, CA 94158 USA

**Keywords:** Neuroscience, Psychology

## Abstract

Early-life stress (ELS) leads to stress-related psychopathology in adulthood. Although dysfunction of corticotropin-releasing hormone (CRH) signaling in the bed nucleus of the stria terminalis (BNST) mediates chronic stress-induced maladaptive affective behaviors that are historically associated with mood disorders such as anxiety and depression, it remains unknown whether ELS affects CRH function in the adult BNST. Here we applied a well-established ELS paradigm (24 h maternal separation (MS) at postnatal day 3) and assessed the effects on CRH signaling and electrophysiology in the oval nucleus of BNST (ovBNST) of adult male mouse offspring. ELS increased maladaptive affective behaviors, and amplified mEPSCs and decreased M-currents (a voltage-gated K^+^ current critical for stabilizing membrane potential) in ovBNST CRH neurons, suggesting enhanced cellular excitability. Furthermore, ELS increased the numbers of CRH^+^ and PACAP^+^ (the pituitary adenylate cyclase-activating polypeptide, an upstream CRH regulator) cells and decreased STEP^+^ (striatal-enriched protein tyrosine phosphatase, a CRH inhibitor) cells in BNST. Interestingly, ELS also increased BNST brain-derived neurotrophic factor (BDNF) expression, indicating enhanced neuronal plasticity. These electrophysiological and behavioral effects of ELS were reversed by chronic application of the CRHR1-selective antagonist R121919 into ovBNST, but not when BDNF was co-administered. In addition, the neurophysiological effects of BDNF on M-currents and mEPSCs in BNST CRH neurons mimic effects and were abolished by PKC antagonism. Together, our findings indicate that ELS results in a long-lasting activation of CRH signaling in the mouse ovBNST. These data highlight a regulatory role of CRHR1 in the BNST and for BDNF signaling in mediating ELS-induced long-term behavioral changes.

## Introduction

Early-life stress (ELS) exposure is a major risk factor for developing psychopathologies in adulthood^1–3^. ELS leads to long-lasting alterations in hypothalamus–pituitary–adrenal (HPA) stress-related parameters^3–6^ and expression of plasticity-related genes, and induces maladaptive affective behaviors in adulthood^7,8^. Here we define maladaptive affective behaviors as behaviors that are not beneficial and are historically associated with human mood disorders. Identifying the neural mechanisms underlying the effects of ELS is therefore important to develop effective treatment strategies.

The bed nucleus of stria terminalis (BNST) connects limbic inputs (amygdala and hippocampus) to the hypothalamus and brain stem, and mediates avoidance behaviors. Corticotropin-releasing hormone (CRH) coordinates the behavioral stress response and is regulated by early-life experiences^6,9,10^. CRH dysregulation results in enhanced vigilance/startle in patients with anxiety or comorbid depression anxiety^11^. CRH is highly expressed in both the hypothalamic paraventricular nucleus (PVN) and in the BNST^12^. In BNST, the highest concentration of CRH neurons is found in the oval nucleus (ovBNST)^12–14^, which connects directly to several limbic nuclei and plays an important role in regulating outflow of information from BNST^15^. BNST CRH neurons, likely through indirect innervation of hypothalamic PVN neurons^16^, are also thought to modulate stress responses^17–19^. We and others have reported that optogenetic stimulation of the ovBNST is sufficient to induce avoidance behaviors^20,21^. We also found that ovBNST CRH dysfunction mediates chronic stress-induced avoidance behaviors in adult mice^21^. Although ELS affects CRH and induces persistent developmental alterations in the hypothalamus and other areas^22–25^, remarkably little is known about how ELS modulates CRH signaling in BNST.

The dam is the primary caregiver of her pups in the laboratory-raised animals. To study long-term effects of ELS, many ELS paradigms mainly target the mother-infant interaction. To better understand how ELS influences the BNST and related maladaptive affective behaviors in adult mice, we exposed newborn mice to 24 h of maternal separation (MS) on postnatal day 3 (PND3). This is a widely used rodent ELS paradigm that resembles elements of maternal neglect and causes maladaptive alterations that last into adulthood^1,4,26,27^, including changes in hippocampal adult neurogenesis and synaptic plasticity, and impaired spatial and fear learning^28^. Here, we used MS to assess the electrophysiological profile of adult ovBNST CRH neurons and characterize expression of CRH signaling, including the upstream regulator pituitary adenylyl cyclase (AC)-activating peptide (PACAP; also known as adenylate cyclase-activating polypeptide 1) and the CRH inhibitor striatal-enriched protein tyrosine phosphatase (STEP; also known as protein tyrosine phosphatase nonreceptor type 5). We focused on miniature excitatory postsynaptic currents (mEPSCs) and M-currents, which are subthreshold voltage-gated, non-inactivating outward K^+^ currents that are critical for stabilizing membrane potential, setting the cellular threshold for action potential firing^29–31^, and regulating synaptic potential-spike coupling^32^. M-current suppression can also augment excitatory synaptic responses^33^. We hypothesized that MS may lead to long-lasting alterations in M-currents, mEPSCs, and BNST CRH signaling that can be observed in adulthood.

In addition, CRH regulates brain-derived neurotrophic factor (BDNF)^34^, a well-known growth factor implicated in neuronal plasticity^35,36^. Notably, BDNF disruption can alter HPA reactivity^37^, and single-nucleotide polymorphism variations in the BDNF gene are associated with individual differences in susceptibility of mood disorders^38,39^. BDNF activation also mediates social stress-induced maladaptive affective and social avoidance behaviors^40,41^ and glucocorticoid-enhanced contextual fear memory^42,43^. As BDNF plays an important role in determining how environmental factors lead to mood disorders^37^, we hypothesized that MS may also lead to long-lasting changes in BNST BDNF expression.

## Materials and methods

### Animals

All procedures were in accordance with National Institutes of Health standards and approved by the Institutional Animal Care and Use Committees of either Rutgers or University of Science and Technology of China. Adult male wild-type C57BL/6J mice were purchased from Jackson Laboratory and bred in-house. In total, 89 adult male mice were used. All animals were maintained under controlled temperature (22 °C) and photoperiod conditions (12 h light/dark cycle; lights on between 6 a.m. and 6 p.m.) with food and water provided ad libitum.

All offspring used (after MS) were male. They were assigned to six different cohorts when they reached 10–12 weeks of age. The first cohort (*n* = 10 for both MS and Control group; each randomly chosen from ten different litters) was used for behavioral assessment followed by immunohistochemistry (each randomly selected *n* = 6 per group). The second cohort (*n* = 6 for both Control and MS group; each randomly chosen from six different litters) was used for ovBNST electrophysiological recordings. Tissue and blood plasma were collected between 9 and 11 a.m. from a third cohort (seven to nine mice in either Control or MS group, randomly chosen from seven to nine different litters) and used for quantitative PCR (qPCR) assessment and plasma corticosterone (CORT) level evaluation.

For electrophysiological recordings testing R121919, R121919 + forskolin, R121919 + BDNF, R121919 + BDNF + GF109203X, or BDNF effects alone, a fourth cohort of Control (*n* = 8) and MS group (*n* = 8) was used (each randomly chosen from eight different litters). Additional cohorts containing Control + R121919 and MS + R121919 group (*n* = 8 per group; each randomly chosen from eight different litters) or MS + R121919 + BDNF group (*n* = 7 per group, each randomly chosen from seven different litters) were used for drug infusions and subsequent behavioral tests.

### MS protocol

MS is based on the absence of maternal care for a period of 24 h, during which the pups are kept warm as previously described^28^. For breeding, one male was housed with two female mice. Pregnant female mice were individually housed at the beginning of the third gestational week and monitored daily. When a newborn litter was observed, the previous day was defined as PND0. Dams were left undisturbed with their litters until PND3. At 9 a.m. of PND3, the dams were removed, placed into a novel cage, and returned to the vivarium. MS litters remained in the home cage and were placed on a heating pad in a separate room. Litters were kept at 28–32 °C during the 24 h separation period. At 9 a.m. on PND4, the dam was returned to the home cage and the whole cage was placed back into the vivarium.

### Behavior

Mice were allowed overnight adaptation in the behavior rooms and behavioral tests were performed between 8 and 12 a.m. Detailed descriptions of sucrose preference test (SPT), elevated plus maze (EPM) test, open-field (OF) test, and novelty suppressed feeding (NSF) tests can be found in the Supplemental Methods^44,45^.

### Plasma CORT measurement

Mice were anesthetized by euthasol (pentobarbital sodium; Henry Schein, NY; 150 mg/kg intraperitoneal) and decapitated. Trunk blood samples were collected and plasma was stored at −80 °C for CORT measurements using an enzyme-linked immunoassay kit according to the manufacturer’s instructions (K014-H1; DetectX, Arbor Assays, MI).

### Real-time quantitative reverse-transcriptase PCR

BNST tissue was dissected and total RNA and protein from the total anterior BNST was extracted. mRNA expression was analyzed with qPCR. Additional details are provided in the Supplemental Methods.

### Western blotting

Protein samples from anterior BNST were probed with anti-BDNF antibody (ab226843; rabbit, 1 : 1000; Abcam, MA). Glyceraldehyde 3-phosphate dehydrogenase (GAPDH) was used as internal control (rabbit, 1 : 10,000; G9545; Sigma-Aldrich, MO). Additional details are in the Supplemental Methods.

### Brain tissue, immunohistochemistry, and image acquisition

Following behavioral tests, 12 mice from the first cohort (*n* = 6 for MS or Control group) were anesthetized and perfused transcardially with 4% paraformaldehyde. Brains were cryoprotected in 30% sucrose before 40 μm-thick sections were cut. Immunohistochemistry was performed using standard procedures, with antibodies anti-CRH (rabbit, ab8901, 1 : 400; Abcam, MA), anti-c-fos (rabbit, 9F6, 1 : 800; Cell Signaling, MA), anti-PACAP (rabbit, ab216627, 1 : 700; Abcam, MA), and anti-STEP (mouse, 23E5, 1 : 500; Novus Biologicals, CO). For CRH staining, colchicine (Sigma-Aldrich; C9754) was intracerebral ventricular-administered 48 h before perfusion. One microliter of colchicine (10 μg/μl in 0.9% saline) was injected into the lateral ventricle (A/P: −0.5 mm; M/L: ±1.0–1.1 mm; D/V: −2.5 mm)^46^ through cannula connected to an UltraMicroPump (UMP3, World Precision Instruments, FL) and SYS-Micro4 Controller (UMC4; World Precision Instruments, FL) at 100 nl/min. Signal amplification was performed with biotinylated goat-anti-rabbit (A27035; Invitrogen) or anti-mouse (A28176; Invitrogen) (both 1:10,000) IgG superclonal secondary antibodies, followed by avidin-biotin complex (PK6100; Vector Laboratories). Chromogen development was performed with DAB (SK-4100; Vector Laboratories; with 0.01% H_2_O_2_). For c-fos/CRH double-immunofluorescence staining, colchicine was administered as described above. Anti-c-fos (goat, sc-52-G, 1 : 500; SantaCruz, CA) and anti-CRH (rabbit, ab8901, 1 : 400; Abcam, MA) primary antibodies were used followed by Alexa Fluor 488 (donkey-anti-goat, A-11055, 1 : 400; Invitrogen, CA) and Alexa Fluor 594 (goat-anti-rabbit, A-11037, 1 : 400; Invitrogen, CA) the next day.

Photographs were taken with an Invitrogen EVOS FL Auto 1 Cell Imaging System (Invitrogen, CA) and numbers of immunopositive cells were manually counted bilaterally at a ×20 magnification. Additional details are described in the Supplemental Methods.

### Electrophysiological recordings

Standard whole-cell voltage-clamp patch recording was performed as previously described^21,47,48^. All drugs used were purchased from Tocris (MN, USA) unless otherwise specified. For additional details, please see the Supplemental Methods.

After a quick decapitation in the morning, coronal BNST slices (250 μm) were cut in 4 °C oxygenated (95% O_2_, 5% CO_2_) high-sucrose artificial cerebral spinal fluid (aCSF) containing (mM): 208 sucrose, 2 KCl, 26 NaHCO_3_, 10 glucose, 1.25 NaH_2_PO_4_, 2 MgSO_4_, 1 MgCl_2_, 10 HEPES pH 7.3, 300 mOsm, and transferred to an auxiliary chamber at room temperature (25 °C; recovery 1–2 h) in standard recording aCSF containing (mM): 124 NaCl, 5 KCl, 2.6 NaH_2_PO_4_, 2 MgCl_2_, 2 CaCl_2_, 26 NaHCO_3_, 10 glucose pH 7.3, 310 mOsm until recording. Single slices were transferred to the recording chamber mounted on an Olympus BX51W1 upright fluorescent microscope and were continually perfused with 35 °C oxygenated aCSF. Targeted neurons were viewed with an Olympus water-immersion lens.

Recordings were performed using glass pipettes pulled with a PC-10 Puller (Narishige, Japan). Axopatch 200B amplifier, Digidata 1322 A Data Acquisition System, and pCLAMP software (version 10.2; Molecular Devices, Sunnyvale, CA) were used for data acquisition and analysis. Input resistance, series resistance, and membrane capacitance were all monitored throughout the experiments. Only cells with a stable series resistance (<30 MΩ; <20% change over the course of the recording) and suitable input resistance (>500 MΩ) were used for analysis.

In total, 23 Control and 22 MS mice were used for the recordings, which were restricted to ovBNST CRH neurons. They were identified based on both anatomical criteria (dorsal location halfway between the tip of lateral ventricle and the top of AC) and by using post hoc immunohistochemical confirmation (labeled with Alexa Fluor 633 dye (green; Life Technologies, CA) in the internal recording solution)^21,47^. Immunohistochemistry was performed with anti-CRH primary antibody (rabbit, 1 : 1000; Abcam, MA) and goat-anti-rabbit Alexa Fluor 594 secondary antibody (red; 1 : 1000; Life Technologies, CA). Overall, the success rate of neurons fulfilling both criteria was ~30%.

To record M-currents, pipettes (3–5 MΩ resistance) were filled with an internal recording solution containing (mM): 10 NaCl, 128 K-gluconate, 1 MgCl_2_, 10 HEPES, 1 ATP, 1.1 EGTA, and 0.25 GTP pH 7.3, 300 mOsm. Tetrodotoxin (TTX) (1 μM) was included in the recording ACSF to block Na^+^-spike-dependent synaptic inputs. Under voltage-clamp, a standard deactivation protocol^21,47^ was used to elicit K^+^ currents during 500 ms voltage steps from −30 to −75 mV in 5 mV increments after a 300 ms prepulse to −20 mV. The amplitude of M-current was measured as determined by the difference between the instantaneous (<10 ms) and sustained current (>475 ms) of the current trace under control conditions (TTX only, 1 μM, 5 min). After 5 min baseline recording, the deactivation protocol was repeated twice and averaged. To examine whether action potential firing is regulated by the M-current, 40 μM XE991 (KCNQ-selective channel blocker) was perfused in the bath solution and firing activity was continuously monitored in current-clamp mode.

To study excitatory synaptic transmission, pharmacologically isolated mEPSCs were recorded^21,47^ with picrotoxin (50 μM) to block GABA_A_ receptor-mediated inhibitory synaptic transmission, D-APV (50 μM) to block NMDA receptor-mediated currents, and TTX (1 μM) to block action potentials. Internal solution (in mM): 40 CsCl, 10 HEPES, 0.05 EGTA, 1.8 NaCl, 3.5 KCl, 1.7 MgCl_2_, 2 Mg-ATP, 0.4 Na_4_-GTP, 10 phosphocreatine, and 5 *N*-(2,6-dimethylphenylcarbamoylmethy)triethylammonium pH 7.3, 280–290 mOsm. After a stable 5 min baseline recording, mEPSCs were continuously recorded for 10 min. The mEPSC properties during the last 5 min were compared between different group conditions.

To test CRHR1-mediated effects, coronal BNST slices from eight MS or eight control mice (each randomly chosen from eight different litters) were incubated with 1 μM CRHR1-selective antagonist R121919^49^ with or without 50 μM forskolin^21^ for 60 min before recording. To test BDNF effects on R121919’s reversal effects, BNST slices from seven MS (randomly chosen from seven different litters) were incubated with 1 μM R121919 together with 100 ng/ml BDNF^50,51^, with or without 3 μM GF109203X^52^ for 60 min before recording. To test BDNF effects, coronal slices from BNST of eight Control mice (randomly chosen from eight different litters) were incubated with 100 ng/ml BDNF^50,51^ with or without 3 μM GF109203X^52^ for 60 min before recording.

### Local cannula drug infusion into the ovBNST

MS or Control mice (*n* = 8 per group) were bilaterally implanted with a guide cannula (C315G/SPC, Plastics One, VA) directly into the ovBNST (bregma AP + 0.2 mm, ML 1.0 mm, DV −4.1 mm). After a 1 week recovery period, R121919 (1 μg in 0.5 μl saline)^53^, with or without the protein kinase A (PKA)-selective agonist forskolin^21^ (120 nM in 0.5 μl saline), was continuously infused at 0.05 μl/min into ovBNST for 7 days. Another group of MS mice (*n* = 7) was infused with R1219191 (1 μg in 0.5 μl saline) together with BDNF (0.375 μg in 0.5 μl saline)^54^ into ovBNST for 7 days. Additional details are in the Supplemental Methods.

### Quantification of surface expressed phosphorylated AMPA-receptor GluR1 (pGluR1-S845)

To compare the surface expression of phosphorylated GluR1 (AMPA-receptor subunit 1 at Serine 845 (S845)), we used brain slice surface biotinylation^55^. Coronal BNST slices (300 μm thickness) from six Control and six MS mice were freshly cut and recovered at 31 °C in oxygenated aCSF (composed of (mM): 125 NaCl, 2.5 KCl, 1.2 NaH_2_PO_4_, 1.2 MgCl_2_, 2.4 CaCl_2_, 26 NaHCO_3_, and 11 glucose) for 40 min. Then, slices were incubated with 0.75 ml sulfo-*N*-hydroxysuccinyl-SS-biotin (Pierce Chemical Company) on ice for 45 min. Slices were then washed with quenching buffer, incubated on ice and then gently pelleted by centrifuge at 200 × *g* for 1 min. Tissue was broken up with 400 μl cold RIPA/PI (RIPA supplemented with 1 μM leupeptin, 1 μM pepstatin, 1 μM aprotinin, and 1 mM phenylmethyl sulfonyl fluoride) and rotated at 4 °C to complete lysis. After the cellular debris was centrifuged at 18,000 × *g* for 15 min at 4 °C, lysate protein fraction was prepared for biotinylation using streptavidin-agarose beads (Thermo Fisher Scientific, IL). SDS-polyacrylamide gel electrophoresis (PAGE) (2×) sample buffer was added and samples were incubated at 4 °C until analysis. After pelleting the beads, biotinylated proteins were eluted. Final samples were then stored frozen. Total lysate samples were thawed and rotated in parallel with bead samples for 30 min. Then proteins were separated on SDS-PAGE gels and identified by immunoblotting with an anti-phospho S845 GluR1 antibody (ab76321; rabbit, 1 : 1000; Abcam, MA). Total GluR1 (ab31232; rabbit, 1 : 700; Abcam, MA) was also assessed as an endogenous control.

### Statistical analysis

Sample size and animal numbers were estimated based on previous studies. Investigators were blind to group allocations for all experiments. All data are presented as mean ± SEM. Statistical analyses were conducted with GraphPad Prism (La Jolla, CA, USA). The normality and variance of data distribution between two groups were analyzed by Kolmogorov–Smirnov test and Levene’s test, respectively (*p* > 0.05). For data that did not conform to normality or homoscedasticity, non-parametric tests were applied. M-current *I*–*V* plots between various groups were compared using a two-way analysis of variance (ANOVA) (with group as between-subject factor, and voltage as within-subject factor, respectively), followed by post hoc Tukey’s comparisons. At each individual voltage (−75 to −25 mV), unpaired two-tailed Student’s *t*-tests were used for comparisons. For mEPSCs, amplitude and frequency were analyzed with Mini Analysis (Synaptosoft, NJ) and compared using unpaired two-tailed Student’s *t*-test. For body weight gain, behavior, immunohistochemical (IHC), and plasma CORT concentration, data were analyzed with a one-way ANOVA and post hoc Tukey’s comparison. *n* represents the number of cells or animals. Differences were considered significant when *p* < 0.05.

## Results

### MS results in adulthood maladaptive affective behaviors

**Fig. 1:**
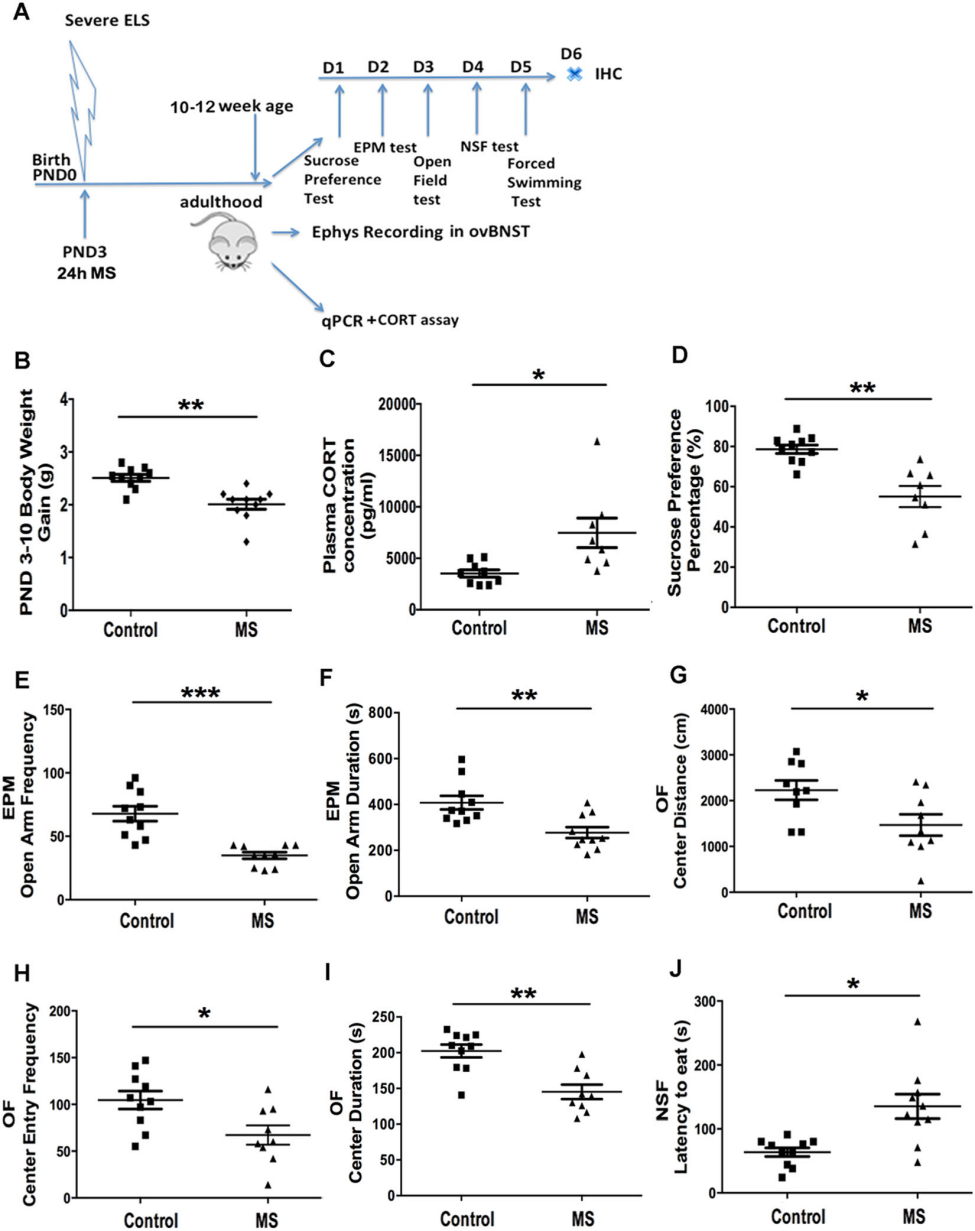
Experimental scheme depicting the early-life stress (ELS) paradigm and a comparison of the negative-valence behavioral outcomes of maternally separated (MS) mice in adulthood. **a** Newborn mice at postnatal day 3 (PND3) were subjected to a severe early-life stress (ELS), consisting of 24 h of maternal separation (MS) from their mother. When they reached adulthood at 10 to 12 weeks of age, they were separated into different cohorts for different experimental tests. For the first cohort, a series of negative-valence behaviors was assessed, including the sucrose preference test (SPT), elevated plus maze (EPM) test, open-field (OF) test, novelty suppressed feeding (NSF) test, and forced swimming test (FST). Mice were then perfused for immunohistochemical (IHC) studies. The second cohort was used for electrophysiological (Ephys) recordings (including M-current and miniature excitatory postsynaptic currents) of CRH neurons of the oval nucleus of the bed nucleus of the stria terminalis (ovBNST). For the third cohort, brains were collected and BNST tissue was extracted for a quantitative PCR (qPCR) study. Blood samples were collected to assess corticosterone (CORT) concentrations. **b** Body weight gain from postnatal day (PND) 3–10 was significantly decreased in the maternal separation (MS) group (*n* = 7) compared to the control (Control) group (*n* = 7). **c** Basal plasma corticosterone (CORT) concentration was significantly increased in the maternal separation (MS) group (*n* = 8) compared to the Control group (*n* = 9). **d** The sucrose preference percentage was significantly decreased in the MS group (*n* = 8) compared to Controls (*n* = 10). **e** Frequency in the open arm was significantly decreased (*p* < 0.001) in MS (*n* = 10) vs. Controls (*n* = 10) mice in the EPM test. **f** Duration in the open arm was significantly decreased (*p* < 0.01) in MS (*n* = 10) vs. Control (*n* = 10) mice in the elevated plus maze (EPM) test. **g** Comparison of the distance that MS (*n* = 9) mice vs. Control (*n* = 10) mice traveled in the center of open-field (OF) test revealed a significant decrease for the MS group (*p* < 0.05). **h** The frequency at which the mice enter into the center of the open-field (OF) was significantly decreased (*p* < 0.05) in MS (*n* = 9) compared to Control (*n* = 10) mice. **i** Duration of the time spend in the center of the open-field (OF) was significantly decreased (*p* < 0.01) in MS (*n* = 9) vs. Control (*n* = 10) mice. **j** The latency to eat food pellets in the novelty suppressed feeding (NSF) test was significantly increased (*p* < 0.05) in the MS (*n* = 9) mice compared with Control (*n* = 10) mice (*n* = 9–10 animals per group; *p < 0.05; **p < 0.01; ***p < 0.001; NS: no significant difference).

We began by exposing PND3 mice to MS (timeline in Fig. 1a). MS-exposed mice gained significantly less body weight from PND3-10 relative to Controls (Fig. 1b; *p* < 0.01). In adulthood, however, no differences in body weights were found between the two groups (Supplemental Fig. S1). MS-exposed adult mice had higher basal plasma CORT levels (Fig. 1c) relative to Controls (*p* < 0.05). MS also induced maladaptive affective behaviors in adulthood. Specifically, sucrose preference was decreased in MS-exposed mice relative to Controls (F(1,16) = 10.313; *p* < 0.01; Fig. 1d). In EPM, MS increased avoidance as measured by decreased open arm entries (Fig. 1e; F(1,18) = 26.44; *p* < 0.001) and open arm duration (Fig. 1f; F(1,18) = 11.760; *p* < 0.01). In OF, MS decreased center distance (F(1,16) = 5.840; *p* < 0.05; Fig.1g), center entry frequency (F(1,17) = 7.090; *p* < 0.05; Fig. 1h), and center duration (F(1,17) = 18.095; *p* < 0.01; Fig. 1i). In NSF, MS increased eating latency (F(1,18) = 12.498; *p* < 0.01; Fig. 1j). Importantly, no differences were found in OF total distance or EPM total entry frequency (Supplemental Fig. S1). MS also did not affect home-cage latency or home-cage food consumption in NSF (Supplemental Fig. S1). PND3 MS did not affect maternal care from PND4-11 (Supplemental Fig. S2). Taken together, MS exposure at PND3 effectively induced maladaptive affective behaviors in adult mice.

### MS activates CRH-associated signaling in the adult ovBNST

**Fig. 2:**
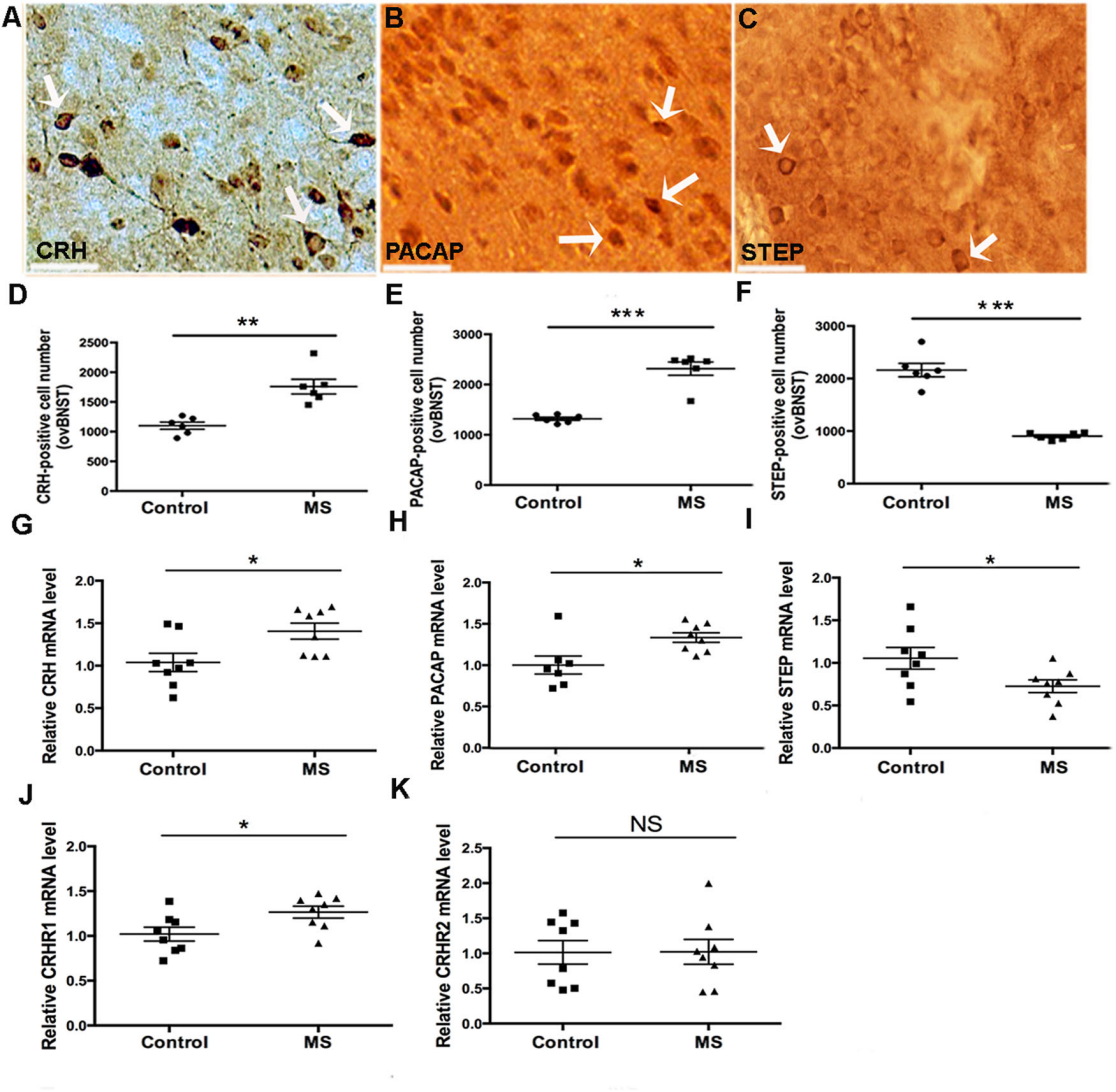
Comparison of CRH, PACAP, and STEP-immunostaining and qPCR comparison of mRNA expression levels of CRH, PACAP, STEP, CRHR1, and CRHR2 in BNST from Control vs. maternally separated (MS) mice. **a** Example of CRH^+^ cells in BNST; white arrows point to typical CRH^+^ cells; scale bar 50 μm. **b** Example of PACAP^+^ cells in BNST; white arrows point to typical PACAP^+^ cells; scale bar 50 μm. **c** Example of STEP^+^ cells in BNST; white arrows point to typical STEP^+^ cells; scale bar 50 μm. **d** Comparison of CRH^+^ cell numbers in ovBNST showed higher number of CRH^+^ cells from MS (*n* = 6) compared to Control mice (*n* = 6). **e** Similarly, a comparison of PACAP^+^ cell number in ovBNST showed higher number of PACAP^+^ cells from MS mice (*n* = 6) compared to Control mice (*n* = 6). **f** On the contrary, comparison of STEP^+^ cell numbers in ovBNST revealed lower numbers of STEP^+^ cells from MS mice (*n* = 6) compared to Control mice (*n* = 6). **g** mRNA expression of CRH was significantly increased in BNST from the MS (*n* = 8) relative to the Control mice (*n* = 8; *p* < 0.05). **h** Similarly, PACAP mRNA expression was higher in the BNST from the MS (*n* = 8) vs. the Control group (*n* = 7) (*p* < 0.05). **i** On the contrary, comparison of STEP mRNA in the BNST revealed lower STEP mRNA levels in the MS (*n* = 8) vs. the Control group (*n* = 8) (*p* < 0.05). **j** CRHR1 mRNA expression was higher in the BNST of the MS (*n* = 8) group compared to the Control (*n* = 9) group of mice (*p* < 0.05). **k** No significant difference was found for mRNA expression of CRHR2 in the BNST from Control (*n* = 8) vs. MS (*n* = 8) mice (*p* > 0.05) (*p < 0.05; **p < 0.01; ***p < 0.001. NS: nonsignificant different (*p* > 0.05)).

CRH is highly expressed in ovBNST (Fig. 2a)^17^. We previously reported that chronic variable mild stress (CVMS) in adult male mice induces maladaptive avoidance behaviors by increasing ovBNST CRH expression^21^; thus, we hypothesized that ELS also activates BNST CRH signaling and alters levels of the upstream activator PACAP (Fig. 2b) and the CRH inhibitor STEP (Fig. 2c). MS increased the number of CRH^+^ cells in ovBNST relative to Controls (F(1,10) = 23.24; *p* < 0.01; Fig. 2d; lower-magnification comparison example shown in Supplemental Fig. S3). In ovBNST of MS mice, PACAP expression was also higher (F(1,10) = 53.68, *p* < 0.001) (Fig. 2e and Supplemental Fig. S3), whereas STEP expression (Fig. 2f and Supplemental Fig. S3) was decreased in ovBNST (F(1,10) = 93.03, *p* < 0.001). Also, MS increased numbers of CRH^+^ cells and PACAP^+^ cells and decreased STEP^+^ cells in the whole anterior BNST (Supplemental Fig. S4). When we quantified the surrounding antero-dorsal region of BNST (adBNST), the numbers of c-fos^+^, CRH^+^, PACAP^+^, and STEP^+^ cells in adBNST were unchanged (Supplemental Fig. S4). Together, these data demonstrate that MS selectively activated CRH signaling in ovBNST.

To complement our IHC results, we next assessed mRNA expression of CRH, PACAP, STEP, and of the CRH receptors CRHR1 and CRHR2 in BNST by qPCR. Similar to our IHC results, MS increased CRH (F(1,14) = 6.642, *p* < 0.05; Fig. 2g) and PACAP mRNA expression (F(1,13) = 7.701, *p* < 0.05; Fig. 2h), but decreased STEP mRNA expression (F(1,14) = 4.991, *p* < 0.05; Fig. 2i) relative to controls. Interestingly, although CRHR1 mRNA expression was increased in the MS group (F(1,14) = 5.856, *p* < 0.05; Fig. 2j), CRHR2 mRNA expression remained unchanged (Fig. 2k).

### MS induces neuronal activation by increasing cellular excitability of CRH^+^ ovBNST neurons

**Fig. 3:**
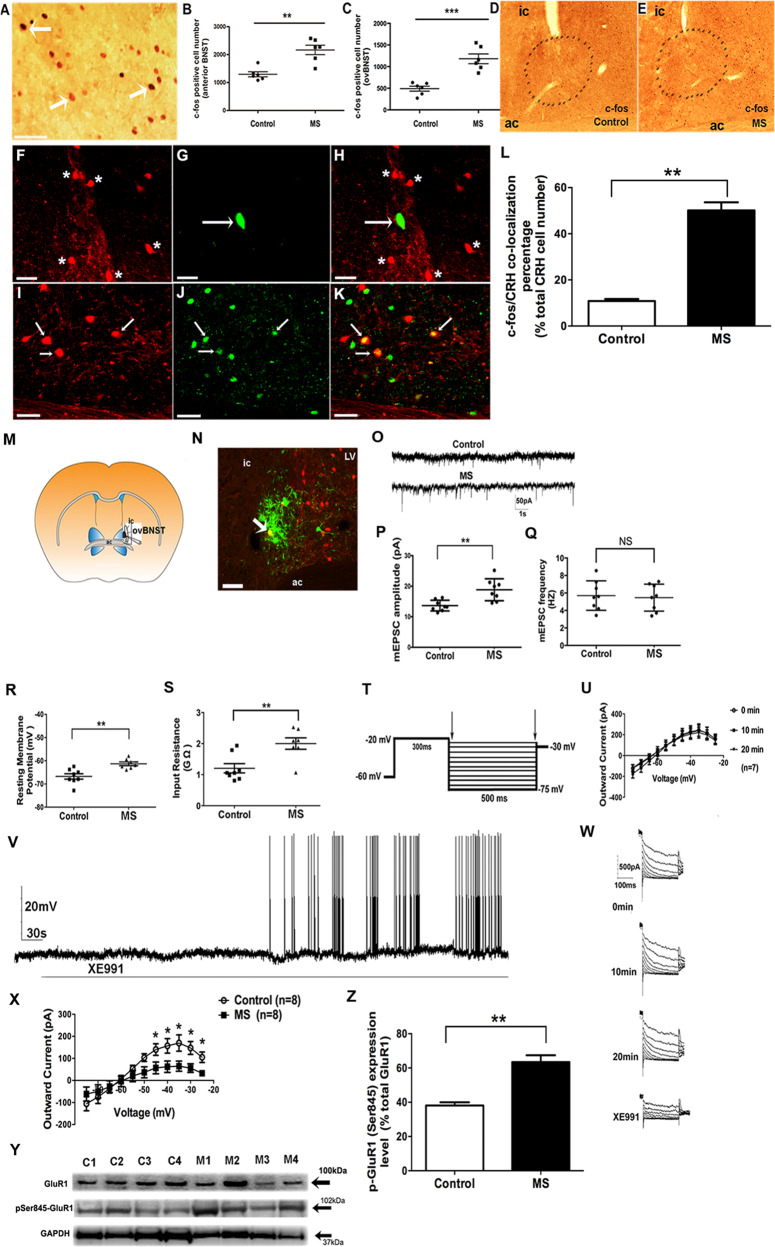
Maternal separation (MS) increased cellular excitability of CRH neurons in the oval nucleus of the bed nucleus of the stria terminalis (ovBNST) of adult mice by inhibiting M-current and potentiating miniature excitatory postsynaptic current (mEPSC). **a** Typical example of c-fos immunopositive cells in BNST; scale bar 50 μm. **b** c-fos immunopositive cell number was significantly increased in the total anterior BNST in MS (*n* = 6) vs. Control (*n* = 6) mice. **c** c-fos cell number was significantly increased in the oval nucleus of BNST (ovBNST) in MS (*n* = 6) vs. Control (*n* = 6) mice. **d** c-fos immunostaining pattern in BNST from Control mice. **e** c-fos immunostaining pattern in BNST from maternally separated (MS) mice. **f** CRH immunostaining (red; pointed by star) in the ovBNST from Control mice. **g** c-fos immunostaining (green; pointed by white arrow) in the ovBNST of Control mice. **h** Lack of colocalization pattern of c-fos/CRH in the ovBNST of Control mice. **i** CRH immunostaining in the ovBNST from MS mice (red; pointed by white arrow). **j** c-fos immunostaining in the ovBNST of MS mice (green; pointed by white arrow). **k** Colocalization pattern of c-fos/CRH in the ovBNST of MS mice (yellow; pointed by white arrow). scale bar: 50 μm. **l** Quantification of average percentage of number of c-fos/CRH colocalized cells in the total number of CRH-immunopositive cells in the ovBNST shows a higher percentage in the MS group of mice (*n* = 5) compared to Control mice (*n* = 5). **m** Example showing the anatomical location of the electrophysiological recording site in the ovBNST (whole-cell patch clamping); ic: internal capsule; ac: anterior commissure. Mice were subjected to a 24 h MS protocol at PND3, after which BNST coronal slices were used for electrophysiological recording at adulthood. **n** A representative example of a CRH^+^ cell in the mice ovBNST after patch clamp recording. Confirmation of the CRH neurochemical profile was achieved by intracellular dye labeling (Alexa Fluor 633, green) followed by immunohistochemical analysis (Alexa Fluor 594, red). White arrow points to a confirmed recorded CRH^+^ neuron (in yellow color) in ovBNST. LV: lateral ventricular. Scale bar 50 μm. **o** Examples of recorded mEPSC traces of ovBNST CRH neurons from Control vs. MS mice. Example trace shows 50 pA and 1 s. **p** Average mEPSC amplitude recorded in the CRH neurons from ovBNST is increased in MS (*n* = 8 cells) compared to Control mice (*n* = 8 cells). **q** For the average mEPSC frequency recorded in CRH neurons from ovBNST, there is no significant difference between MS (*n* = 8 cells) and Control cells (*n* = 8 cells). **r** Comparison of cellular resting membrane potential (RMP) in CRH neurons in ovBNST from Control (*n* = 8 cells) vs. MS mice (*n* = 7 cells) revealed a depolarized RMP in the MS mice. **s** Comparison of cellular input resistance (IR) in ovBNST CRH neurons between Control mice (*n* = 8 cells) vs. MS mice (*n* = 7 cells) revealed a higher IR in the MS mice. **t** The deactivation protocol used to record the M-current. From a holding potential of −60 mV, a voltage jump to −20 mV (300 ms) is followed by steps from −30 to −75 mV in 5 mV increments (500 ms). **u** Examples of the *I*–*V* curve of the M-current allow comparison of recordings at 0, 10, and 20 min, and shows the M-current does not run down during 20 min recording (*n* = 7 cells). **v** An example of a continuous action potential firing activity trace recorded under current-clamp mode in an ovBNST CRH neuron during bath perfusion with the selective KCNQ/Kv7 channel blocker XE991 (40 μM). Robust firing bursts were found after around 5 min XE991 application. **w** Example of M-current traces shown at 0, 10, and 20 min, and after subsequent perfusion with KCNQ-selective channel blocker XE991 (bath incubation, 40 μM). M-currents were robustly suppressed after XE991 application. **x** *I*–*V* plot shows diminished outward M-current in ovBNST CRH neurons in the MS mice ranging from −75 to −25 mV compared to Control mice (each *n* = 8 cells). A significant difference was found in the voltage range between −45 and −25 mV. **y** Example for comparison purposes of a western blotting showing protein bands of pSer845-GluR1 (MW = 102 kDa) in BNST tissue of Control mice (lane 1–4; C1–C4) vs. MS mice (lane 5–8; M1–M4). Total GluR1 (MW = 100 kDa) was used as the internal control. GAPDH (MW = 37 kDa) was also shown. **z** Representative graph showing an increase in relative percentage of expression level of p-GluR1 (Ser845) in BNST tissue from CVMS (*n* = 5 mice) vs. Control (*n* = 5 mice) mice (*p* < 0.01) normalized with total GluR1 expression. (*p < 0.05**;** **p < 0.01; ***p < 0.001; NS nonsignificant different (*p* > 0.05).

We next explored the cellular mechanism underlying activation of BNST CRH signaling by MS. We hypothesized that MS would increase cellular excitability of the CRH^+^ neurons in ovBNST. To this end, we first examined c-fos (following handling; Fig. 3a) as a marker of neuronal activation. c-fos immunoreactivity was significantly elevated in both the anterior BNST (F(1,10) = 20.78, *p* < 0.01) and ovBNST (F(1,10) = 30.04, *p* < 0.001) of MS mice (Fig. 3b–e), indicating increased neuronal activation. By contrast, in adBNST, the numbers of c-fos^+^ cells were not different between MS and control mice (Supplemental Fig. S4).

Given the increases in both c-Fos activation and CRH signaling in BNST after MS, we next performed double-immunostaining of c-fos (Fig. 3g, j) and CRH (Fig. 3f, i) in the ovBNST to compare expression patterns. As shown in Fig. 3f–k, we observed an increase in c-fos^+^/CRH^+^ cells in the ovBNST of MS (white arrows; Fig. 3k) relative to control mice (Fig. 3h; white star and arrow, lack of colocalized cells). Quantification demonstrated that a higher percentage of c-fos^+^/CRH^+^ cell number relative to the total CRH^+^ cell number was found in the MS group (50.1 ± 3.5%, *n* = 5 mice; *p* < 0.01) vs. Control group of mice (10.8 ± 0.9%, *n* = 5 mice) (Fig. 3l).

Next, we evaluated cellular excitability by recording individual ovBNST neurons (Fig. 3m) that were CRH^+^ in ex vivo adult slices. Figure 3n shows a typical CRH^+^ ovBNST neuron (in yellow; designated by a white arrow). We first measured excitatory glutamatergic neurotransmission (representative traces shown in Fig. 3o) in CRH^+^ ovBNST neurons. Interestingly, the average mEPSC amplitude was increased (Fig. 3p) in MS-exposed mice (*t* = 3.679, *p* < 0.01), but no changes in mEPSC frequency were observed (Fig. 3q). Furthermore, MS led to a significantly depolarized resting membrane potential (RMP) (Fig. 3r; *t* = 3.877, *p* < 0.01) and an increased input resistance (Fig. 3s; *t* = 3.366, *p* < 0.01) in adulthood. Our result is consistent with previous findings in hypothalamic CRH neurons of neonatal ELS mice^56^.

To next determine the underlying mechanism of the enhanced excitability, we examined M-currents (KCNQ/Kv7 channels)^30,32,33^. Based on the mEPSC results, we hypothesized that MS would alter the M-currents. Using a standard deactivation–activation protocol (Fig. 3t), maximum M-currents were recorded at −35 mV (Fig. 3u) and no rundown was observed over 20 min (Fig. 3u, w) in CRH^+^ ovBNST neurons. When firing activity of ovBNST CRH neurons was monitored in current-clamp mode, we found KCNQ/Kv7-selective channel blocker XE991 induces robust action potential burst firing after 5–6 min (Fig. 3v), demonstrating a tonic inhibitory role of M-currents in setting neuronal excitability. As shown in Fig. 3w, M-current amplitude was robustly inhibited by XE991. Interestingly, outward M-current was attenuated in MS mice (Fig. 3x), especially at higher voltages (*p* = 0.043, 0.041, 0.030, 0.021, 0.016 at −45, −40, −35, −30, and −25 mV, respectively; *n* = 8 cells per group), with a repeated measures effect of MS (F(1,14) = 9.858, *p* = 0.007). Thus, MS at PND3 induces long-lasting increases in cellular excitability and a hyperactivation of CRH neurons in the ovBNST in adulthood.

Our qPCR data suggested that ELS induces long-lasting activation of CRHR1. CRHR1 is a Gs-coupled receptor linked to PKA activation and PKA regulates membrane trafficking of the AMPA-receptor GluR1 subunit via direct phosphorylation of the intracellular carboxy terminal motif at S845^57^. Interestingly, we also found a significant increase in the surface expression of pS845-GluR1 (Fig. 3y) in the BNST of MS mice when normalized to the total GluR1: (63.5 ± 4.0% of total GuR1) compared to Control mice (38.1 ± 1.9% of total GuR1; *p* < 0.01) (Fig. 3z). Total BNST GluR1 was not different between MS and Control mice (Supplemental Fig. S5). These results confirmed that the increased mEPSC amplitude in the MS group of mice is caused by an increased phosphorylation of surface GluR1.

### CRHR1 antagonist R121919 application to adult ovBNST reverses MS effects on behavior and neurophysiology, and R121919 effects are abolished by co-administration of the PKA agonist forskolin

**Fig. 4:**
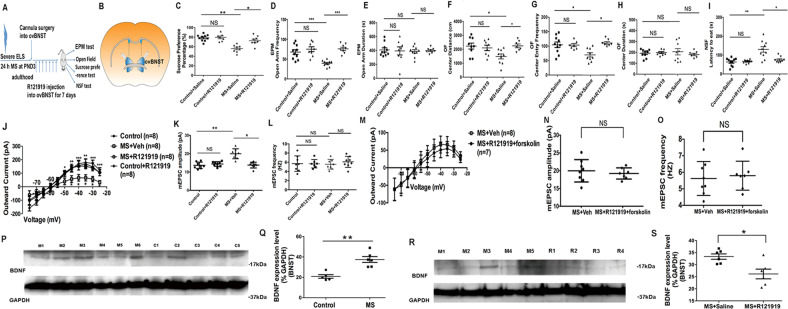
CRHR1-selective antagonist R121919 infused into ovBNST reverses the maladaptive electrophysiological and affective behavioral effects of maternal separation (MS). These were abolished by co-incubation with forskolin. An increased expression level of BDNF was found in BNST from maternally separated (MS) mice, which was normalized by local in vivo application of the CRHR1-selective antagonist R121919 into ovBNST. **a** Scheme showing that the CRHR1-selective antagonist R121919 (1 μg dissolved in 0.5 μl saline) was chronically and bilaterally infused into ovBNST of adult MS mice through a cannula for a continuous period of 7 days to compare its effects on maladaptive affective behaviors in the EPM test, OF test, SPT test and NSF test. **b** Anatomical example shows the location of the cannulas that were bilaterally inserted into ovBNST. **c** The sucrose preference percentage was significantly increased in MS + R121919 mice (*n* = 7) compared to MS + Saline mice (*n* = 10). **d** Duration; time spent in the open arm in the EPM was not significantly changed in MS + R121919 (*n* = 7) compared to MS + Saline mice (*n* = 10). **e** The frequency of open arm entries in the EPM test was significantly increased in MS + R121919 (*n* = 7) compared to MS + Saline mice (*n* = 10). **f** Duration of time spend in the center of the OF was not significantly different in MS + R121919 (*n* = 7) relative to MS + Saline mice (*n* = 10). **g** Distance traveled in the center of OF was significantly increased in MS + R121919 mice (*n* = 7) compared to MS + Saline mice (*n* = 10). **h** Frequency of center entries in the OF was significantly increased in MS + R121919 mice (*n* = 7) compared to MS + Saline mice (*n* = 10). **i** The latency to pellet consumption in the NSF was significantly decreased in MS + R121919 mice (*n* = 7) compared to MS + Saline mice (*n* = 10). **j** Pre-incubation of BNST brain slices from MS mice with 10 μM CRHR1-selective antagonist R121919 (*n* = 7 cells) for 60 min significantly normalized the decreased outward M-current in CRH neurons from MS mice incubated with Vehicle (*n* = 8 cells) compared to Control mice (*n* = 8 cells). R121919 had no effect on Control slices (*n* = 8 cells each condition). **k** The mEPSC amplitude was significantly decreased in R121919 pre-incubated cells (*n* = 7 cells) compared to Vehicle-treated cells from MS mice (*n* = 8 cells). R121919 has no effect on Control slices (*n* = 8 cells each condition). **l** mEPSC frequency has no significant change in R121919 pre-incubated cells (*n* = 7 cells) compared to untreated cells from MS mice (*n* = 8 cells). R121919 also has no effect on Control slices (*n* = 8 cells each condition). **m** R121919’s reversal effect on the M-current was abolished by co-incubation with forskolin in BNST slices from MS mice with Vehicle treated (*n* = 8 or 7 cells each condition). **n** R121919’s reversal effect on the mEPSC amplitude was abolished by co-incubation with forskolin in BNST slices from MS mice with Vehicle treated (*n* = 8 cells each condition). **o** R121919 had no effect on the mEPSC frequency when co-incubated with forskolin in BNST slices from MS mice with Vehicle treated (*n* = 8 or 7 cells each condition). **p** Example of a western blotting showing protein bands representing BDNF (MW = 17 kDa) in BNST tissue of MS (lane 1–5; M1–M5) vs. Control mice (lane 6–10; C1–C5). GAPDH (MW = 37 kDa) was used as the internal control. **q** Representative graph showing an increased relative percentage of BDNF expression in BNST tissue from MS (*n* = 6 mice) vs. Control (*n* = 5 mice; *p* < 0.01) mice. **r** Example of western blotting showing protein bands of BDNF (MW = 17 kDa) in BNST tissue from MS mice with chronic R121919 infusion into their ovBNST for a continuous 7 days (lane 6–9; R1–R4) vs. MS mice (lane 1–5; M1–M5). GAPDH (MW = 37 kDa) was used as the internal control. **s** Representative graph showing an increased relative percentage of BDNF expression in BNST tissue from MS + R121919 (*n* = 6 mice) compared with MS + Saline (*n* = 5 mice; *p* < 0.01) mice. *p < 0.05; **p < 0.01; ***p < 0.001; NS not significantly different).

We next investigated whether the ELS-induced maladaptive affective phenotype is mediated by CRHR1 in BNST. To this end, the CRHR1 antagonist R121919 (1 μg, dissolved in 0.5 μl saline) was infused into ovBNST continuously for 7 days (Fig. 4a, b) prior to behavioral testing. Chronic R121919 infusion reversed the effects of MS on sucrose preference: [Fig. 4c; F(3,29) = 14.561, *p* < 0.001; MS + Saline vs. MS + R121919, *p* < 0.01;], EPM open arm entry frequency: [Fig. 4d; F(3,32) = 13.266, *p* < 0.001; MS + Saline vs. MS + R121919, *p* < 0.001], OF center distance: [Fig. 4f; F(3,29) = 3.727, *p* < 0.05; MS + Saline vs. MS + R121919, *p* < 0.01], and OF center entries: [Fig. 4g; F(3,29) = 5.756, *p* < 0.01; MS + Saline vs. MS + R121919, *p* < 0.01]. R121919 also normalized NSF latency: [Fig. 4i; F(3,31) = 18.889, *p* < 0.001; MS + Saline vs. MS + R121919, *p* < 0.01]. R121919 did not affect EPM open arm duration (Fig. 4e) or OF center duration (Fig. 4h). Importantly, R121919 had no effects on these behavioral parameters in Control mice (Control + Saline vs. Control + R121919; *p* > 0.05).

When R121919 (1 μM) was pre-incubated with BNST slices, the decrease in MS-induced M-current of CRH neurons in ovBNST was also reversed (Fig. 4j), with significant effects of group (F(3,27) = 27.773, *p* < 0.05) and voltage (F(10,54) = 115.56, *p* < 0.001). M-currents were also restored in MS + R121919 slices at all higher voltages (*p* = 0.026, 0.006, 0.001, 0.003, 0.002, and 0.001 at −50, −45, −40, −35, −30, and −25 mV, respectively) compared with MS + Vehicle slices.

Similarly, for mEPSC amplitude, a significant group effect was found (F(3,28) = 19.802, *p* < 0.05) and mEPSC amplitude was reversed in the MS + R121919 group compared with MS + Vehicle group (*p* < 0.01; Fig. 4k). mEPSC frequency was not changed (Fig. 4l). Importantly, R121919 had no effects on these electrophysiology measures in Control slices (Fig. 4j–l). Collectively, these results demonstrate that the CRHR1-selective antagonist R121919 reverses the maladaptive effects of MS on behavior and BNST electrophysiology, indicating that BNST CRHR1 is a major mediator of the long-lasting effects of MS in adult mice.

As CRHR1 is a Gs-coupled membrane receptor linked to PKA activation, we next tested whether MS effects on electrophysiology persist when CRHR1 is blocked but PKA is still activated. MS BNST slices were pre-incubated ex vivo with R121919 (1 μM) and forskolin (50 μM) for 60 min before recording. Interestingly, no significant difference in M-currents was found between MS + Vehicle vs. MS + R121919 + forskolin slices (Fig. 4m) and neither were differences found in mEPSC amplitude (Fig. 4n) or frequency (Fig. 4o) between these two groups. Collectively, these results indicate that the effects of MS on BNST electrophysiology and behavior (Supplemental Fig. S6) persist when CRHR1 is blocked but PKA is activated. This suggests PKA activation likely acts as a downstream effector that mediates MS maladaptive effects.

### MS increases BDNF protein expression in BNST

Increased excitability of ovBNST CRH neurons reflects an adaptation of BNST neuronal plasticity. Importantly, BDNF is crucial for establishing neuronal plasticity^36^ and BDNF expression in a variety of brain regions is sensitive to ELS exposure^58–60^. As CRHR1 activation induces BDNF activation^34^ and a chronic variable stress paradigm increases BDNF expression in BNST^61^, we next investigated whether BDNF expression in BNST was altered after MS.

Interestingly, increased levels of BDNF expression were found in BNST of MS (Fig. 4p; 37.49 ± 2.87% of GAPDH, *n* = 6) vs. Control mice (Fig. 4q; 20.84 ± 1.76% of GAPDH, *n* = 5; *p* = 0.001). Blocking CRHR1 with R121919 decreased BDNF expression in the BNST (Fig. 4r) of MS + R121919 mice (26.15 ± 2.02% of GAPDH, *n* = 6) relative to the MS + Saline group (Fig. 4s; 33.36 ± 1.11% of GAPDH, *n* = 6; *p* = 0.015), suggesting that the increase in BDNF expression in MS mice is CRHR1-dependent.

### MS effects persist when R121919 and BDNF are co-infused into ovBNST

**Fig. 5:**
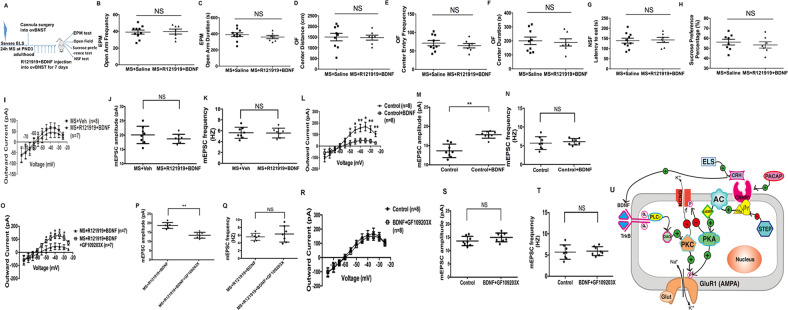
MS effects on maladaptive behaviors and BNST neurophysiological parameters persist in the presence of BDNF even when CRHR1 is blocked. Maladaptive neurophysiological effects of BDNF were abolished when co-incubated with PKC-selective antagonist GF109203X in BNST slices. **a** Scheme showing that CRHR1-selective antagonist R121919 together with BDNF was chronically and bilaterally infused through a cannula into ovBNST of CVMS mice for 7 days. Then maladaptive behaviors were compared (including EPM test, OF test, SPT test, and NSF test). **b** Open arm duration time of the EPM from MS+Saline mice (*n* = 10) compared to R121919 + BDNF-treated MS mice (*n* = 7). **c** Frequency that mice entered into the open arm of the EPM after infusion of R121919 + BDNF into ovBNST of CVMS mice (*n* = 7) compared to saline-infusions in MS mice (*n* = 10). **d** Distance traveled in the center of the open-field (OF) after infusion of R121919+BDNF into ovBNST of MS mice (*n* = 7) compared to saline-infused MS mice (*n* = 10). **e** Duration of time spent in the center of OF test after infusion of R121919+BDNF into ovBNST of MS mice (*n* = 7) compared to saline-infusion in CVMS mice (*n* = 10). **f** Frequency of entries in the OF center after infusion of R121919+forskolin into ovBNST in MS mice (*n* = 7) compared to saline-infusion in MS mice (*n* = 10). **g** Latency to pellet consumption in the NSF test after infusion of R121919 + BDNF into ovBNST of CVMS mice (*n* = 7) compared to saline-infusion in MS mice (*n* = 10). **h** Sucrose preference percentage after infusion of R121919 + BDNF into ovBNST in MS mice (*n* = 7) compared to saline-infusion in MS mice (*n* = 10). **i** Effect of pre-incubation of BNST slices from MS mice with 60 min R121919 + BDNF (*n* = 7 cells) on the M-current outward amplitude in ovBNST compared to vehicle-treated neurons from MS mice (*n* = 8 cells). **j** Pre-incubation of BNST slices from MS mice with 60 min R121919 + BDNF (*n* = 7 cells) compared to vehicle-treated neurons from MS mice (*n* = 8 cells). **k** Pre-incubation of BNST slices from MS mice with 60 min R121919 + BDNF (*n* = 7 cells) compared to vehicle-treated neurons from MS mice (*n* = 8 cells). **l** *I*–*V* plots of M-current after 100 μg/ml BDNF application (*n* = 8 cells) showed a decreased M-current (from −50 to −25 mV) compared with Controls (*n* = 8 cells). **m** Average mEPSC amplitude after 100 μg/ml BDNF application (*n* = 8 cells) was increased compared with Control cells (*n* = 8 cells). **n** Average mEPSC frequency after 100 μg/ml BDNF application (*n* = 8 cells) did not change compared to Control cells (*n* = 8 cells). *p < 0.05; **p < 0.01; ***p < 0.001; NS: nonsignificant different (*p* > 0.05). **o** PKC-selective antagonist GF109203X abolished the M-current suppression effect of BDNF when co-incubated with BDNF together with R121919 in BNST slices from MS-exposed mice. **p** GF109203X reversed the amplified mEPSC amplitude when co-incubated with BDNF together with R121919 in BNST slices from MS-exposed mice. **q** GF109203X had no effect on the mEPSC frequency when co-incubated with BDNF in BNST slices from MS-exposed mice. **r** BDNF has no effect on the M-current when co-incubated with GF109203X in BNST slices from Control mice. **s** BDNF has no effect on the mEPSC amplitude when co-incubated with GF109203X in BNST slices from Control mice. **t** BDNF has no effect on the mEPSC frequency when co-incubated with GF109203X in BNST slices from Control mice. **u** Our cellular model proposes that early-life stress (ELS) induces CRH production and BDNF release in BNST, then (1): CRH sequentially activates CRHR1, a Gs-protein coupled membrane receptor, which is linked to AC activation on the cell membrane (adenylyl cyclase; coupled to CRHR1). Activation of Gαs then triggers cAMP production, which in turn activates the PKA enzyme. Activation of PKA is thought to initiate two parallel phosphorylation pathways: phosphorylation of the KCNQ channel on the cellular membrane to mediate inhibition of the M-current; and phosphorylation of GluR1 subunit of AMPAR on the postsynaptic membrane increases its surface expression to mediate potentiation of mEPSC amplitude. The neuropeptides PACAP (which functions as an upstream stress regulator) and STEP (functions as a CRH inhibitor) function to activate and inhibit CRH, respectively, in the CRH-associated stress signaling network. Meanwhile, (2) BDNF production lies downstream of CRHR1. BDNF can activate the TrkB receptor on the cell membrane, which in turn activates phospholipase C (PLCϒ) to catalyze hydrolysis of membrane-bound PIP_2_ into IP_3_ and DAG; DAG then activates PKC to activate its downstream phosphorylation of KCNQ channel subunit and phosphorylation of GluR1 subunit on the postsynaptic membrane, which together mediate M-current inhibition and potentiated mEPSC amplitude. These two CRHR1-PKA and BDNF-PKC-mediated, parallel pathways can function independently and subsequently mediate suppression of the M-current and amplification of mEPSC amplitude, respectively.

BDNF signaling in the nucleus accumbens mediates maladaptive affective and social avoidance behaviors induced by chronic social defeat stress in adult mice^40,62^, so we next asked whether increases in BDNF expression in BNST underlie the maladaptive affective phenotype induced by MS. We specifically tested whether MS effects persist when CRHR1 is blocked during BDNF infusion. Interestingly, when 0.75 μg/μl of BDNF was chronically co-administered in vivo with R121919 (1 μg) into the ovBNST (Fig. 5a), the maladaptive affective behaviors induced by MS persisted. When MS mice infused with Saline were compared with MS mice that had R121919 and BDNF co-infused, no differences were found in EPM open arm entry frequency (Fig. 5b) and duration (Fig. 5c), OF center distance (Fig. 5d), center entries (Fig. 5e), and center duration (Fig. 5f). There also was no difference in NSF latency (Fig. 5g) or sucrose preference (Fig. 5h) between the two groups (all *p* > 0.05; MS + Saline vs. MS + R121919 + BDNF).

We next pre-incubated ex vivo BNST slices from MS mice with R121919 (1 μM) and BDNF (100 ng/ml) together for 60 min, and recorded M-currents and mEPSCs. No difference in M-currents (Fig. 5i), mEPSC amplitude (Fig. 5j), or mEPSC frequency (Fig. 5k) was found between MS + Vehicle and MS + R121919 + BDNF slices. Thus, maladaptive MS effects persist when CRHR1 is blocked but BDNF is present.

### Bath application of BDNF mimics MS effects on M-currents and mEPSCs in ovBNST CRH neurons

We next investigated whether BDNF in the BNST is sufficient to directly mediate the maladaptive cellular effects of MS. BDNF increases glutamatergic transmission in the entorhinal cortex^51^, so we tested whether exogenous application of BDNF (100 ng/ml) in control mice could mimic maladaptive effects of MS. Similar to effects of MS, M-currents were suppressed by BDNF application (Fig. 5l; F(1,14) = 5.233, *p* = 0.038; and *p* = 0.018, 0.012, 0.005, 0.012, 0.008, and 0.009 at −50, −45, −40, −35, −30, and −25 mV, respectively). BDNF also increased mEPSC amplitude relative to Control (Fig. 5m; *n* = 8 per group; *p* < 0.01), without altering mEPSC frequency (Fig. 5n). Taken together, exogenous BDNF application suppresses M-currents and increases mEPSC amplitude, mimicking MS effects. These data indicate that the presence of BDNF in the BNST is sufficient to mediate maladaptive cellular effects of MS.

### BDNF effects on CRH M-currents and mEPSCs are abolished by PKC antagonism

BDNF activates TrkB receptors, which triggers PLCγ activation and subsequent hydrolysis of PIP_2_ into IP_3_ and DAG, which in turn activates protein kinase C (PKC). As activated PKC phosphorylates KCNQ channels^63,64^ and GluR1 receptors^52,65^, we next investigated whether the maladaptive cellular effects of BDNF are mediated by PKC activation.

To this end, we pre-incubated ex vivo BNST slices from MS mice with either R121919 (1 μM) and BDNF (100 ng/ml), or R121919 (1 μM), BDNF (100 ng/ml), and the PKC-selective antagonist GF109203X (3 μM) for 60 min. Interestingly, the MS/BDNF-induced decrease in M-currents was reversed in MS + R121919 + BDNF + GF109203X slices compared to MS + R121919 + BDNF slices (Fig. 5o; F(1,12) = 7.734, *p* = 0.016). Moreover, the mEPSC amplitude was also normalized (Fig. 5p; *p* < 0.01) and there was no difference in mEPSC frequency (Fig. 5q). Interestingly, the maladaptive affective behavioral phenotype of MS + R121919 + BDNF was also reversed by blocking PKC (Supplemental Fig. S7). Therefore, BDNF likely acts through PKC to mediate the maladaptive affective effects of MS. Interestingly, the PKC-selective antagonist GF109203X (3 μM) also abolished the effects of BDNF alone on ovBNST M-currents (Fig. 5r) and mEPSC amplitude (Fig. 5s). mEPSC frequency (Fig. 5t) was also unchanged, relative to Control when incubated with GF109203X. Therefore, we propose a model showing ELS- > CRH- > CRHR1- > PKA, and ELS- > BDNF- > PKC signaling cascades in Fig. 5u.

## Discussion

ELS increases the risk to develop stress-related mood disorders in adulthood, results in long-lasting transcriptional alterations^66,67^ and permanently changes several behavioral and neuroendocrine stress responses^68^. To mimic elements of early maternal neglect, we applied 24 h MS at PND3. Adult male mice that were exposed to ELS displayed maladaptive affective behaviors, increased BNST CRH signaling, and enhanced CRH neuronal excitability in adulthood. Here we define “maladaptive” as “behavior that is not beneficial and that is historically associated with human mood disorders”. Therefore, severe ELS lead to an enduring CRH dysfunction in the adult BNST.

### ELS induces long-term maladaptive affective behaviors

MS, which reprograms HPA function^4^, is commonly used in rodents to study ELS. We found that MS at PND3 increased basal plasma CORT and induced typical maladaptive affective behaviors in adulthood. Compared with other protocols (such as limited bedding and/or nesting material or fragmented maternal care)^69,70^, the MS paradigm we used is more severe^8,36^. The decrease in body weight gained between PND3-10 in MS mice (Fig. 1b) may reflect a temporary period of under-nutrition, caused by absence of the dam, who provides important tactile and nutritional input to the developing brain during this period^71^. Also, maternal signals directly affect the development of the emotional circuitry and cognitive performance of the offspring^72^. As we made sure the pups were kept warm and no differences in overall maternal behavior towards the pups were observed between PND4-11 (Supplemental Fig. S2), the combination of a lack of maternal sensory and nutritional inputs for 24 h likely comprises the major elements of ELS that account for the long-term negative outcomes.

### ELS results in hyperexcitation of ovBNST CRH neurons

PACAP is a key upstream regulator of CRH stress signaling^73^, whereas STEP selectively buffers CRH neurons against overactivation to mediate stress resilience^74^. Both PACAP and STEP colocalize with CRH in ovBNST (Supplemental Fig. S8). In addition, CRHR1 also colocalized with STEP (Supplemental Fig. S9) and CRH (Supplemental Fig. S10) in the ovBNST. ELS-induced increases in PACAP and decreases in STEP expression indicate an imbalance in CRH signaling in BNST. Based on these results, we propose that increased BNST CRH signaling is a key mediator of the long-lasting maladaptive behaviors.

ELS-induced mEPSC amplitude increases in ovBNST CRH neurons are indicative of enhanced glutamatergic neurotransmission, likely due to a postsynaptic effect^75^ originating from increased phosphorylation of GluR1 subunits and membrane surface distribution (Fig. 3y, z)^76^. Other ELS paradigms have also implicated glutamate receptor subunits in cognitive changes, indicating an important role for these receptors in ELS effects^77,78^. We found that ELS activated CRHR1 (a Gs-coupled receptor linked to the AC-cAMP-PKA pathway). We thus defined an underlying mechanism whereby PKA-mediated phosphorylation of GluR1 and KCNQ channels is critical for maladaptive effects of ELS (Figs. 4m–o and 5u). In addition, M-current suppression (Fig. 3x) per se could augment excitatory synaptic responses^33^. Diminished M-currents enhance intrinsic excitability^32^ and allow for a more rapid firing^29^, as shown by XE991 application (Fig. 3v). Consistently, ELS mice displayed a depolarized cellular RMP (Fig. 3r). In addition, the increased c-fos/CRH double-stained cells (Fig. 3l) directly demonstrate enhanced activation of ovBNST CRH neurons by ELS. Taken together, ELS results in significant hyperactivation of ovBNST CRH neurons.

How the ELS-induced overexcitation of CRH neurons in the ovBNST results in long-lasting maladaptive affective behaviors remains unknown. One possible cause could be a disrupted BNST neuronal circuitry. We and others found that acute optogenetic stimulation of the adult ovBNST increases maladaptive avoidance behaviors^20,21^. The BNST is an important node in the limbic forebrain stress-response circuitry that transmits stress information from structures such as the amygdala and hippocampus to the PVN and thereby participates in regulation of HPA axis responsivity^16^. ELS results in persistent structural and functional changes in many structures^71,79^, including the prefrontal cortex^80^, hippocampus^81^, amygdala^82^, and other cortical areas^11^. Through both direct and indirect connections^83,84^, these regions form a circuit that controls avoidance behaviors^79^. Specifically, ELS results in smaller PFC volumes and poor executive functioning^85^, increases in amygdala volume^86^, and reductions in hippocampal volume^87^. ELS also increases connectivity between the mPFC and amygdala^88^, and causes abnormal hippocampus–amygdala–prefrontal cortex connections^89^. This altered circuitry likely contributes to ELS-induced maladaptive affective behaviors. Importantly, the BNST is a critical node of the avoidance circuitry^90^, which has mutual projections to the central amygdala^53^, PVN^91^, ventral tegmental area (VTA)^92^ and lateral hypothalamus^93^, whereas it further receives input from the ventral subiculum^94^ that is involved in HPA-negative feedback. Although ELS effects on the hippocampus^7^, dorsal raphe^23^, and VTA^95^ are well-described, little is known about how ELS affects the BNST. Here we provide data implicating that a hyperactivate CRH circuitry in the ovBNST can result in increased HPA axis activation. The exact details of how BNST dysfunction induces this maladaptive affective phenotype will require future studies at the circuitry level.

### ELS-induced CRHR1-PKA and BDNF-PKC signaling converge to hyperactivate ovBNST CRH neurons

Our data that ELS-induced maladaptive affective behaviors are reversed by application of the CRHR1 antagonist R121919 is consistent with a previous report^96^ and indicates that CRHR1 in BNST may be a novel molecular target for therapeutic interventions. Indeed, in the ovBNST, we found many CRHR1 cells to colocalize with CRH (shown in Supplemental Fig. S10). CRHR1 activation initiates a stress response^97^, whereas CRHR2 facilitates stress recovery^98^. Although R121919 reverses the neurophysiological changes seen in ELS mice, this effect was abolished by BDNF co-administration. Also, BDNF infusions in the BNST of naïve mice mimicked ELS effects. Consistently, CRH can directly upregulate BDNF transcription (de novo synthesis) through CRHR1-cAMP-PKA signaling in cerebellar neurons^34^. Future studies are necessary to investigate whether R121919 also reverses MS-induced changes in expression of CRH, PACAP, STEP, and CRHR1.

BDNF activation in the VTA-NAc circuitry promotes stress susceptibility^41^ and facilitates stress-induced maladaptive affective behaviors^40^. Interestingly, social stress-induced BDNF increases in the NAc are also mediated by CRH^41^. We found that the maladaptive cellular effects of BDNF are reversed by GF109203X application (Fig. 5o–t), suggesting that downstream PKC activation is necessary^63,64^. Therefore, BDNF-induced PKC activation likely drives the maladaptive effects of MS when CRHR1 is blocked.

Our findings thus suggest activation of parallel pathways of CRHR1-PKA and BDNF-PKC signaling by ELS. This parallel regulation of BNST CRH neurophysiology by CRHR1-PKA and BDNF-PKC signaling converges to suppress M-currents and amplify mEPSC amplitude (Fig. 5u). Enhanced GluR1 conductance^99^ and increased GluR1 synaptic delivery^52^ results in increased synaptic transmission^65^, eventually triggering neuronal hyperactivity. Thus, our data demonstrate a novel positive feed-forward amplification cascade in the BNST that is triggered by MS.

Our current study also has several limitations. First, we cannot exclude MS-induced changes in other electrophysiological parameters than M-currents and mEPSCs. Second, the BNST is a sexually dimorphic brain structure^100,101^ and, although we focused on male mice in this study, future studies on females are essential. In addition, possible differences in the consequence of phosphorylation by PKA and PKC activation will require future study. Finally, parallel changes in other brain areas likely have also contributed to the maladaptive effects of ELS.

In summary, we report that a severe early-life adverse experience, lasting only 24 h at PND3, results in long-lasting maladaptive changes in ovBNST function in adulthood. Our findings highlight that dysregulation of CRHR1-BDNF signaling in BNST underlies, at least in part, ELS-related maladaptive affective aspects of behavioral disorders. Future in-depth investigations of the limbic circuitry (e.g., by examining simultaneous electrophysiological response at several key nodes within such circuitry) will help to understand the full extent of the underlying mechanisms.

## Supplementary information

Supplemental Text

Supplemental Figure 1

Supplemental Figure 2

Supplemental Figure 3

Supplemental Figure 4

Supplemental Figure 5

Supplemental Figure 6

Supplemental Figure 7

Supplemental Figure 8

Supplemental Figure 9

Supplemental Figure 10
