# Supplementary material for: Early-life stress alters affective behaviors in adult mice through persistent activation of CRH-BDNF signaling in the oval bed nucleus of the stria terminalis
Source: Transl Psychiatry. 2020 Nov 11;10:396. doi: 10.1038/s41398-020-01070-3 (PMC7658214; doi:10.1038/s41398-020-01070-3)
Supplement: Supplementary file 1 — Supplemental Text [file 41398_2020_1070_MOESM1_ESM.doc]

**Supplemental Files:**

**Supplemental Methods:**

**Animals:**

The 1^st^ cohort of mice (n=10 for each MS and Control group) was used for negative valence behavioral assessment, including: sucrose preference test (SPT), elevated plus maze (EPM), open field (OF) and novelty suppressed feeding (NSF). Behavioral tests were performed between 0800 and 1200 h under white light. Details are described as below.

**Sucrose Preference Test:**

Animals were first trained to drink 1% (w/v) sucrose solution for a 48 h adaptation period. At 8:30 am in the morning of testing day, mice were given free access to two bottles (each containing normal water and sucrose solution respectively). To avoid left/right preference, the left-right placement order of water vs. sucrose bottle was alternated for each mouse during the middle time-point of the testing period. Bottles were weighed at the beginning and end of the testing 24 h later. The percentage of sucrose solution relative to the total liquid consumed during 24 h was then determined as a measure for anhedonia.

**Elevated Plus Maze:**

The EPM test evaluates anxiety-like behavior [^1^](#_ENREF_1). Mice were placed in the central arena of a black plus-shaped maze, facing an open arm and were left to explore for 10 min. The duration and frequency of which open or closed arms were explored was analyzed by video camera and processed by EthoVision (Noldus, Wageningen, The Netherlands).

**Open-field Test:**

Open-field test analyzes spontaneous exploratory activity and curiosity to novel environment [^2^](#_ENREF_2) and is often used to evaluate anxiety-like behavior together with the EPM test. Open field apparatus consists of a black floor area (40.5 cm × 40.5 cm), with a 37.5 cm high transparent wall. Mice were placed in the center of apparatus (center square) and monitored for 30 min. Data was collected and processed with EthoVision (Noldus, Wageningen, The Netherlands). Distance, duration of time that mice spent in the center, and the frequency of mice entry into the center area was documented and analyzed.

**Novelty Suppressed Feeding (NSF) Test:**

The NSF test elicits competing motivations between the drive to eat and the fear of venturing into the center of a brightly lit arena, and is often used to evaluate anxiety [^1^](#_ENREF_1). Mice were first food-deprived for 18 h (starting at 2:00pm the previous day) and then placed into holding cages at 8:00 am on the testing day. After 60 min, the mice were placed into a novel, brightly lit (1200 lux) arena (16” × 20”) with a pellet of chow placed in the center of the arena affixed to a circular platform of white filter paper (10 cm). The testing arena is covered with 2 cm wooden bedding in the plastic box. Mice were placed in the testing arena for a total of 6 min. The time taken for the mice to bite the food pellet for the first time was recorded as the latency to eat, at which point the pellet was immediately removed from the arena.

**Maternal Behaviors Monitoring:**

Maternal behaviors towards the pups were monitored between 9:00 am and 11:00 am per day, from PND 4 until PND 11, with observation sessions lasting 48 min [^3^](#_ENREF_3). Activity of the dams was scored every 3^rd^ minute, resulting in 16 one-minute epochs per observational session. Scored behaviors include: licking and grooming behavior; nursing behavior; and the time that dam spent off the pups.

**Real-time quantitative RT-PCR analysis:**

After anesthetized by euthasol (pentobarbital sodium; Henry Schein, NY, USA; 150 mg/kg i.p.), the third cohort of MS mice was quickly decapitated and BNST tissue was dissected and frozenly mounted on glass (anatomy from AP +0.10 mm to -0.46 mm). Anterior BNST was punched from 160 μm thick sections and stored at -80°C. Total RNA and protein was extracted using the RNA/Protein purification plus kit (48200; Norgen Biotek CORP, ON, Canada) and stored at -80°C. Each group (Control or MS) contains BNST tissue samples from 7-9 mice from 7-9 different litters. CRH, PACAP and STEP, CRHR1 and CRHR2 mRNA expression was analyzed with eukaryotic 18S rRNA endogenous control (Taqman VIC^TM^ probe; Invitrogen, CA, USA) as control for PCR amplification. cDNA was synthesized using SuperScript™ IV VILO™ Master Mix (with ezDNase™ enzyme) (11766050; Invitrogen, CA, USA) as the reverse transcriptase. Q-PCR was performed with Taqman Fast Advanced Master Mix (4444557; ThermoFisher Scientific, CA, USA), control probe and each target gene FAM Taqman Probe. The relative amount of target gene was calculated using the 2^-ΔΔCt^ method [^4^](#_ENREF_4).

**Western blot:**

Total protein of punched anterior BNST tissue was extracted with the RNA/Protein purification plus kit (48200; Norgen Biotek CORP, ON, Canada) and stored at -80°C. Each group (Control or MS or MS+R121919) contains protein samples from 5-6 mice (randomly selected from 5-6 different litters). Then protein samples were probed by immunoblotting with anti-BDNF antibody (ab226843; rabbit, 1:1000; abcam, MA), and then horseradish peroxidase (HRP)-conjugated goat-anti-rabbit IgG secondary antibodies (1:10,000; 31460, Thermo Fisher Scientific, Waltham, MA) on an iBind Flex Western device (Thermo Fisher Scientific, Waltham, MA). GAPDH was used as internal control (rabbit, 1:10,000; G9545; Sigma-Aldrich, Saint Louis, MO). Immunoblot signals were then detected with ECL chemiluminescence system (SuperSignal West Pico chemiluminescence Substrate, Pierce, IL) and imaged with myECL imager (Thermo Fisher Scientific, Waltham, MA). Immunoblots were quantified and density was calculated using ImageJ software (Wayne Rasband, NIMH, Bethesda, MD). Results were expressed as percentage of GAPDH.

**Electrophysiological Recordings:**

Mice were quickly decapitated at 10:00–11:00 A.M. In total, 23 Control mice and 22 MS mice were used. All the drugs used in the patch clamp recordings were purchased from Tocris (MN, USA) unless otherwise specified.

The brain was rapidly removed from the skull, and a block containing the BNST was immediately dissected and submerged in cold (4°C) oxygenated (95% O2, 5% CO2) high-sucrose artificial cerebrospinal fluid (aCSF) containing (in mM): 208 sucrose, 2 KCl, 26 NaHCO3, 10 glucose, 1.25 NaH2PO4, 2 MgSO4, 1 MgCl2, 10 HEPES; adjusted to pH 7.3, 300 mOsm [^5-7^](#_ENREF_5). Coronal slices (250 μm) were cut on a vibratome at 4°C. The slices were then transferred to an auxiliary chamber in which they were kept at room temperature (25°C) (recovery for 1-2 h) in aCSF consisting of the following (in mM): 124 NaCl, 5 KCl, 2.6 NaH2PO4, 2 MgCl2, 2 CaCl2, 26 NaHCO3, 10 glucose (adjusted to pH 7.3, 310 mOsm) until recording. A single slice was transferred to the recording chamber mounted on an Olympus BX51W1 upright microscope. The slice was then continually perfused with warm (35°C), oxygenated aCSF at 1.5 ml/min. Targeted neurons were viewed with an Olympus 40x water-immersion lens. Standard whole-cell voltage-clamp patch recording procedures and pharmacological applications were performed. Recordings of BNST slices were performed using pipettes made of borosilicate glass and pulled with a PC-10 Puller (Narishige, Japan). Axopatch 700B amplifier, Digidata 1322A Data Acquisition System, and pCLAMP software (version 10.2; Molecular Devices, Sunnyvale, CA) were used for data acquisition and analysis.

To record M-currents, pipettes (3-5 MΩ resistance) were filled with internal solution containing (in mM): 10 NaCl, 128 K-gluconate, 1 MgCl2, 10 HEPES, 1 ATP, 1.1 EGTA, and 0.25 GTP. Internal solution was adjusted to pH 7.3, 300 mOsm. 1 μM TTX was included in the recording ACSF to block Na+-spike–dependent synaptic inputs. Under voltage clamp, a standard deactivation protocol was used to measure K^+^ currents elicited during 500-ms voltage steps from –30 to –75 mV in 5-mV increments after a 300-ms prepulse to –20 mV. From this protocol, the input resistance was determined from the slope of the I-V plot in the range between –60 and –80 mV. Input resistance, series resistance, and membrane capacitance were all monitored throughout the experiments. Only cells with stable series resistance (< 30 MΩ; < 20% change over the course of the recording) and suitable input resistance (> 500 MΩ) were used for analysis. The amplitude of M-current relaxation or deactivation was measured as the difference between the initial (< 10 ms) and sustained current (> 475 ms) of the current trace under control conditions (TTX only; 1 μM; 5 min). After baseline recording (~5 min), deactivation protocol was repeated twice and averaged for analysis.

To study excitatory synaptic transmission in ovBNST neurons, pharmacologically isolated mEPSCs were recorded as described [^6^](#_ENREF_6). Picrotoxin (PTX, 50 μM) was added to block inhibitory synaptic transmission mediated by GABAA receptor, and meanwhile D-APV (50 μM) was added to block currents mediated by NMDA receptors. In addition, TTX (1 μM) was included to block action potential. Internal solution contained (in mM): 40 CsCl, 10 HEPES, 0.05 EGTA, 1.8 NaCl, 3.5 KCl, 1.7 MgCl2, 2 Mg-ATP, 0.4 Na4-GTP, 10 phosphocreatine, and 5 N-(2,6-Dimethylphenylcarbamoylmethy)triethylammonium, and was adjusted to pH 7.3, 280-290 mOsm. After a stable baseline recording of approximately 5-min period, mEPSC was continuously recorded for around 10 min. The mEPSC properties recorded during the last 5 min period were compared between different group conditions.

**Immunofluorescence Staining:**

To assess whether CRH cells co-localize with PACAP or STEP in the ovBNST, double immunofluorescence stainings were performed. The following antibodies and conditions were used: CRH (rabbit, ab8901, 1:400; Abcam, MA), PACAP (mouse, sc-166180, 1:200; Santa Cruz, TX) and STEP (mouse, sc-23892, 1:400; Santa Cruz, TX). After permeabilized with 0.1% Triton for 5 min, mounted BNST sections were washed with TBS for 5 min, then blocked in 10% normal goat serum and 0.3% Triton for 20 min, and then incubated in primary antibodies diluted in 2% normal goat serum and 0.3% Triton at room temperature for 1 h, then incubated overnight at 4 °C. The next day sections were washed and incubated for 2 h in Alexa Fluor Plus 555 (goat-anti-rabbit, A32732, 1:400; Invitrogen, CA), or FITC (goat-anti-mouse, A16079, 1:400; Invitrogen, CA).

To test whether CRHR1 co-localize with STEP in the ovBNST, double immunofluorescence stainings were performed. The following antibodies and conditions were used: CRHR1 (goat, PA5-1880, 1:200; ThermoScientific, MA) and STEP (mouse, sc-23892, 1:250; Santa Cruz, TX). After permeabilized with 0.1% Triton for 5 min, mounted BNST sections were washed with TBS for 5 min, then blocked in 10% normal goat serum and 0.3% Triton for 20 min, and then incubated in primary antibodies diluted in 2% normal goat serum and 0.3% Triton at room temperature for 1 h, then incubated overnight at 4 °C. The next day sections were washed and incubated for 2 h in Alexa Fluor Plus 488 (donkey-anti-goat,1:400; Invitrogen, CA), or Alexa Fluor Plus 594 (goat-anti-mouse, A16079, 1:400; Invitrogen, CA).

After brief rinse, sections were embedded in the ProLong diamond antifade mountant (P36961; Invitrogen, CA). Fluorescent signals were detected and photographs were collected using Invitrogen EVOS FL Auto 2 Cell Imaging System (Invitrogen, CA).

**Local cannula drug infusion into ovBNST:**

For drug infusions through local cannula, a Hamilton syringe (HSYR-1 Syringe 86200, Plastics1, Roanoke, VA) was connected with a segment of PE tubing (C313CT/PKG, Plastics1, Roanoke, VA).

MS or Control mice (n=8 per group) were bilaterally implanted with a guide cannula (C315G/SPC, Plastics One, VA) directly into ovBNST (bregma AP +0.2 mm, ML 1.0 mm, DV -4.1 mm) (anatomy example picture shown in Fig. 6B). After 1 week recovery, R121919 (1μg in 0.5 μl saline) [^8^](#_ENREF_8) with or without PKA-selective agonist forskolin (120 nM in 0.5 μl saline) was continuously infused at a rate of 0.05 μl/min into the ovBNST for 7 days. Another group of MS mice (n=7) was infused with R1219191 (1μg in 0.5 μl saline) together with BDNF (0.375μg in 0.5 μl saline) into ovBNST for 7 days.

**Quantification of surface expression of phosphorylated AMPA-receptor GluR1 (pGluR1-S845):**

To compare the surface expression of phosphorylated GluR1 (AMPA-receptor subunit 1 at Serine 845), a brain slice surface biotinylation method was used to isolate surface pGluR1-SS845. Coronal BNST slices (300 μm thickness) from n=6 Control and n=6 MS mice were freshly cut with vibrating microtome and allowed to recover at 31 °C in oxygenated ACSF (composed of 125 mM NaCl, 2.5 mM KCl, 1.2 mM NaH2PO4, 1.2 mM MgCl2, 2.4 mM CaCl2, 26 mM NaHCO3 and 11 mM glucose) for 40 min. Then slices were incubated with 0.75 ml sulfo-N-hydroxysuccinyl-SS-biotin (sulfo-NHS-SS-biton; Pierce Chemical Company) on ice for 45 min. After being washed with ice cold ACSF, slices were then incubated on ice for 10 min with ice cold ACSF. Then slices were then washed 3 times with ice-cold slice quench buffer (ACSF supplemented with 100 mM glycine) and incubated with 0.75 ml slice quench buffer on ice for 25 min to quench free sulfo-NHS-SS-biotin. Then slices were transferred to microcentrifuge tubes to be gently pelleted by centrifuge at 200 ×g, 1min. After adding 400 μl cold RIPA/PI (RIPA supplemented with 1 μM leupeptin, 1 μM pepstatin, 1 μM aprotinin and 1 mM phenylmethyl sulfonyl fluoride), tissue was broken up by pipetting through P200 pipette. Then dissociated slice/RIPA was transferred to fresh tube, then incubated for 30 min at 4°C and rotated to complete lysis. After cellular debris were pelleted by centrifuging at 18,000 ×g 15 min at 4°C, lysate protein was prepared for biotinylation with streptavidin-agarose beads (Thermo Scientific, IL) according to the protocol [^11^](#_ENREF_11).

Cell lysates were distributed to the tubes containing agarose beads (Thermo Scientific, IL), then additional RIPA/PI were added to bring to samples to a 200 μl volume. Then tubes were rotated and mixed overnight at 4 °C. Equivalent total lysate volume was dispensed in separated tubes for each sample. Then an equal volume of 2× SDS-PAGE sample buffer was added and samples were incubated at 4 °C in parallel with bead samples until samples were analyzed.

After pelleting beads by centrifuging at 18,000 ×g for 2 min, supernatant was aspirated and beads were washed with 0.75 ml RIPA. Then after the final wash, the RIPA was aspirated and biotinylated proteins were eluted from streptavidin beads by reducing the disulfide linkage with 25 μl 2× SDS-PAGE sample buffer and rotating for 30 min. The final samples were stored frozen. Total lysate samples were thawed and rotated in parallel with bead samples for 30 min at room temperature. Then proteins were separated on SDS-PAGE gels and identified by immunoblotting with anti-phospho S845 GluR1 antibody (ab76321; rabbit, 1:1000; abcam, MA).

**Statistics:**

All data are presented as mean ± S.E.M. Statistical analyses were conducted with GraphPad Prism (La Jolla, CA, USA). M-current *I-V* plots between various groups were compared using a two-way ANOVA (with *group* as between subject factor and *voltage* as within subject factor respectively), followed by *post-hoc* Tukey comparisons. At each individual voltage (–75 to –25 mV), unpaired two-tailed Student’s t-tests were used for comparisons. For mEPSCs, amplitude and frequency were analyzed with Mini Analysis (Synaptosoft, NJ) and compared using unpaired two-tailed Student’s *t*-test. For body weight gain, behavior, IHC, qPCR and western blot comparison and plasma CORT concentration, data were analyzed with a one-way ANOVA and *post-hoc* Tukey comparison. For the maternal behavior data, each group consists of data monitored from n=10 litters. Maternal behavior (licking and grooming behavior; nursing behavior; and the time that dam spent off the pups) data were compared between MS vs. Control groups using a two-way ANOVA (with *group* as between subject factor and *postnatal day* as within subject factor respectively), followed by *post-hoc* Tukey comparisons.

*n* represents number of cells or animals or litters. Differences were considered significant when p<0.05.

**Supplemental Results:**

**1.** **Comparison of body weight at PND70, total traveling distance in the OF test and total entry frequency in the EPM test, latency to eat and food consumption amount in the home cage in the NSF test between Control vs. maternal separated (MS) mice when they reached adulthood:**

When the mice reached adulthood at postnatal day 70 (PND70), comparison of body weight reveals no significant difference between Control group (26.04±0.46 g; n=10) and maternal separated (MS) group (26.54±0.39 g; n=10; p=0.42) (Fig.S1A).

In the open field (OF) test, no significant difference in the total distance that mice travelled was found between the two groups (11828.3±744.7 cm, n=10 in Control group vs. 12248.4±1102 cm, n=9 in MS group) (ANOVA F(1,17)=0.103; p=0.757) (Fig.S1B). In the elevated plus maze (EPM) test, no significant difference in the total entry frequency was found between the two groups either (85±6.28, n=10 in Control group vs. 78.4±5.38, n=10 in MS group) (ANOVA F(1,18)=0.637; p=0.435) (Fig.S1C). Taken together, these results indicate that MD mice demonstrated no overall changes in their locomotor activity compared with Control mice.

In the novel suppression feeding test (NSF), no significant difference in the latency to eat in the home cage also was found between the 2 groups: 20.7±2.9 s, n=10 in Control group vs. 20.7±2.5 s, n=10 in MS group; ANOVA F(1,18)=0.000, p=1.00 (Fig.S1D). No significant difference in the food consumption amount in the home cage also was found between the 2 groups: (0.20±0.02 g, n=10 in Control group vs. 0.21±0.02 g, n=10 in MS group) (ANOVA F(1,18)=0.101, p=0.754) (Fig.S1E).

**2. Comparison of the dam’s maternal care behaviors towards the pups between MS vs. Control group of mice from postnatal day 4 to 11 (PND4-11).**

When we compare the dam’s maternal behaviors within this time period, we found MS did not affect: either 1) maternal licking and grooming behavior between MS (n=10) vs. Control (n=10) group of mice (Fig.S2A) (F(1, 17)=43.28, p>0.05); or 2) total nursing time of the dam compared between MS (n=10) vs. Control (n=10) mice (Fig.S2B) (F(1, 17)=59.85, p>0.05); or 3) the time that the dam is leaving off her pups compared between MS (n=10) vs. Control (n=10) mice (Fig.S2C) (F(1, 17)=79.53, p>0.05).

Together, these results indicate that the dam’s overall maternal care behavior did not significantly differ between MS vs. Control mice during the critical postnatal period PDN4-11.

**3. Comparison of immunostaining pattern of c-fos, CRH, PACAP and STEP in the antero-dorsolateral region of BNST (BNSTadl) between mice from Control vs. maternal seprived (MS) group:**

Typical example of immunostaining figures of c-fos (Control Fig. S3A vs. MS Fig. S3B), CRH (Control Fig.S3C vs. MS Fig.S3D), PACAP (Control Fig. S3E vs. MS Fig. S3F) and STEP (Control Fig.S3G vs. MS Fig.S3H) in the antero-dorsolateral BNST (BNSTadl) was compared between Control vs. MS mice and shown in Fig. S3. Arrows point to typical example of immunopositive cells for c-fos (Fig.S3A-B), CRH (Fig. S3C-D), PACAP (Fig.S3E-F) and STEP (Fig.S3G-H). Black dots highlight the border for the oval nucleus of BNST (ovBNST) region within BNSTadl.

**4. Comparison of immunopositive cell numbers for c-fos, CRH, PACAP and STEP in the antero-dorsal region of BNST (adBNST; surrounding ovBNST within BNSTadl) between mice from Control vs. maternal separated (MS) group:**

MS increased the number of CRH^+^ cells in the anterior BNST relative to Controls (anterior BNST: F(1,10)=32.38, p<0.01; Fig.S4A); PACAP expression was also higher in the anterior BNST (F(1,10)=81.31, p<0.001; Fig.S4B) in the MS mice; whereas STEP expression (Fig.S4C) was decreased in the anterior BNST (F(1,10)=42.93, p<0.001) in the MS mice.

When we further subdivide the total anterior BNST into ovBNST and the closely surrounding antero-dorsal region of BNST (adBNST; Fig.S4D), and further compared c-fos, CRH, PACAP and STEP immunopositive cell numbers between Control vs. MS group of mice, we found for c-fos, there is no significant difference in the cell number in the adBNST between Control (805±101) vs. MS group (987±125) (F(1,10)=1.29; p=0.283) (Fig. S4E). Also, no significant difference in the CRH^+^ cell number in the adBNST was found between Control (1235±60) vs. MS group (1278±151) (F(1,10)=0.071; p=0.798) (Fig.S4F). Likewise, for PACAP^+^ cell number in the adBNST, there is no significant difference between Control (1472±111) vs. MS group (1697±108) (F(1,10)=2.113; p=0.177) (Fig. S4G). Again, no significant difference was found in the STEP^+^ cell number in the adBNST compared between the two groups (Control: 1126±77; MS: 1012±76) (F(1,10)=1.099; p=0.319) (Fig.S4H). Together, these data indicate that CRH signaling is selectively activated in the ovBNST region after MS.

**5. Total GluR1 protein expression level is not different in the BNST of Control vs. MS group of mice:**

To complement the pS845-GluR1 result, we also quantified and compared the total GluR1 protein expression level in the BNST of Control vs. MS group of mice. Surprisingly, no significant difference was found between the two groups: Control 45.3±3.1% of GAPDH vs. MS 45.4± 3.0% of GAPDH (Fig.S5; p>0.05), indicating only the phosphorylation of Ser845 but not the total GluR1 amount is affected by MS.

**6. Co-infusion of PKA-selective agonist Forskolin into ovBNST abolished the reversing behavioral effect of R121919:**

Since our Fig.4 shows that the effects of MS on BNST electrophysiology parameters persist when CRHR1 is blocked but PKA is simultaneously activated, we next aimed to investigate whether the reversing effect of the CRHR1-selective antagonist R121919 on the negative-valence behaviors of MS mice was also blocked when PKA-selective agonist forskolin is co-infused into ovBNST together with R121919. Therefore, forskolin (120 nM; dissolved in 0.5 μl saline) was co-administered with R121919 (1 μg; dissolved in 0.5 μl saline) into ovBNST for 7 days.

In the EPM test, there is no significant difference in the open arm duration (Fig.S6A) between MS group (377.11±27.81s) vs. MS+R121919+forskolin group (394.40±21.61s); (F(1,15)=0.207, p=0.656). Again, no significant difference in the EPM open arm entry frequency (Fig.S6B) was found between the two groups: MS group (34.9±2.60) vs. MS+R121919+forskolin group (40.14±3.07); (F(1,15)=1.695, p=0.213).

In the OF test, no significant difference in the center duration (Fig.S6C) was found between the two groups: MS group (189.66±52.37 s) vs. MS+R121919+forskolin group (216.49±23.45 s); (F(1,14)=0.180, p=0.678). Also, no significant difference in the center travel distance (Fig.S6D) was found between the two groups: MS group (1468.89±233.00 cm) vs. MS+R121919+forskolin group (1686.18±165.51 cm); (F(1,14)=0.514, p=0.485). Again, no significant difference in the center entry frequency (Fig.S6E) was found between the two groups: MS group (67.22±10.29) vs. MS+R121919+forskolin group (77.14±6.36); (F(1,14)=0.582, p=0.458).

For the NSF test, similarly, no significant difference in the latency to eat was found between the two groups (Fig.S6F): MS (135.3±19.11 s) vs. MS+R121919+forskolin (125±8.81 s); (F(1,15)=0.181, p=0.676).

Again, for the sucrose preference test, no significant difference in the sucrose preference (Fig.S6G) was found between the two groups: MS group (47.99±6.01%) vs. MS+R121919+forskolin group (54.49±3.20%); (F(1,15)=0.710, p=0.413).

Taken together, when CRHR1 is blocked and PKA is simultaneously activated, the reversing effect of R121919 on the negative-valence behaviors of MS mice was abolished when forskolin is co-infused with R121919 into ovBNST. Therefore, PKA activation likely acts as a final effector downstream of CRHR1 to mediate the maladaptive behavioral effects of MS.

**7**. **BDNF’s maladaptive behavioral effects were abolished when co-infused with PKC-selective antagonist GF109203X:**

Since our Fig.5 shows that the maladaptive electrophysiological effect of MS+R121919+BDNF on ovBNST CRH neurons was reversed when PKC is blocked, we therefore next tested whether the similar effect exist for the maladaptive behavioral effects.

When the PKC-selective antagonist GF109203X (50 ng) [^15^](#_ENREF_15) was co-infused with BDNF (0.375 μg) and R121919 (1 μg; all dissolved in 0.5 μl saline) [^8^](#_ENREF_8) into the ovBNST of MS mice for 7 days, nearly all of the maladaptive behavioral effects of BDNF were abolished:

In the EPM test, there is a significant increase in the EPM open arm duration (Fig.S7A) from MS+R121919+BDNF+GF109203X group (489.6±24.04 s) compared with MS+R121919+BDNF group (359.82±19.87 s; F(1,12)=21.573, p<0.01). Also, significant increase in the EPM open arm entry frequency (Fig.S7B) was found in the MS+R121919+BDNF+GF109203X group (59.71±2.71) compared with MS+R121919+BDNF group (39.71±2.94; F(1,12)=25.04, p<0.001).

In the OF test, significantly increased center duration (Fig.S7C) was found in the MS+R121919+ BDNF+GF109203X group (267.9±23.17 s) compard with MS+R121919+BDNF group (189.1±25.53 s; F(1,12)=10.44, p=0.041). Again, significantly increased center distance (Fig.S7D) was found in the MS+R121919+ BDNF+GF109203X group (2202.43±191.49 cm) compared with MS+R121919+BDNF group (1475.61±116.43 cm; F(1,12)=10.518, p=0.007). Again, significantly increased center entry frequency (Fig.S7E) was found in the MS+R121919+ BDNF+GF109203X group (87.71±6.72) compared with MS+R121919+BDNF group (64.14±6.02; F(1,12)=6.831, p=0.023).

For the NSF test, similarly, less latency to eat (Fig.S7F) was found in the MS+R121919+ BDNF+GF109203X group (105.29±6.39 s) compared with MS+R121919+BDNF group (142.57±12.51 s; F(1,12)=7.041, p=0.021).

Again, for the sucrose preference test (Fig.S7G), significantly higher sucrose preference percentage was found in the MS+R121919+BDNF+GF109203X group (68.53±4.04%) compared with MS+R121919+BDNF group (53.28±3.15%; F(1,12)=8.853, p=0.012).

Taken together, when CRHR1 is blocked by R121919 in the MS mice, the maladaptive behavioral effects of co-infused BDNF on the negative-valence behaviors were abolished when PKC-selective antagonist GF109203X is present in the ovBNST, indicating the maladaptive behavioral effects of BDNF indeed requires PKC activation. Therefore, BDNF likely acts through PKC to mediate its maladaptive behavioral effects.

**8.Immunofluorescence shows co-localization of CRH with both PACAP and STEP in the ovBNST:**

Since previous study has shown STEP and CRH immunoreactivity co-localize in the neurons of the oval nucleus region in the rat BNST (ovBNST) [^12^](#_ENREF_12), we next sought to confirm the expression pattern of these two neuropeptides in the mice ovBNST by immunofluorescence study. Consistently, we found almost complete co-localization of CRH with STEP in the ovBNST (shown in Fig.S8A-C, CRH in red and STEP in green color). Average percentage of co-localized STEP^+^/CRH^+^ double-immunopositive cell number in the total number of CRH^+^ cells is around 63.8%.

Since high density of PACAP expression was found in the ovBNST , and since PACAP and CRH peptide signaling is suggested to be integrated to regulate BNST activity, we sought to further explore the expression pattern of these two neuropeptides in the ovBNST. Similarly, a high percentage of co-localization of CRH with PACAP neuropeptides was also found in the ovBNST (shown in Fig.S8D-F, CRH in red and PACAP in green color). Average percentage of co-localized PACAP^+^/CRH^+^ double-immunopositive cell number in the total number of CRH^+^ cells is around 75%.

Together, our results show that both STEP and PACAP shows a pattern of highly co-localization with CRH in the mice ovBNST.

**9**. **Immunofluorescence shows co-localization of CRHR1 with STEP in the ovBNST:**

STEP is acting as an inhibitory peptide to inhibit CRH function. Since our previous study suggests CRH acts through its receptor CRHR1 in the ovBNST (Hu 2020 JNS; Kash TL and Winder DG 2008 JNS), we hypothesize that STEP may co-localize with CRHR1 in the ovBNST to exert its function. As expected, most CRHR1 cells (green; pointed by the arrows; Fig.S9A) do co-localize with STEP (red; pointed by the arrows; Fig.S9B) in the ovBNST (shown in Fig.S9C); the average percentage of co-localized STEP^+^/CRHR1^+^ double-immunopositive cell number in the total number of CRHR1^+^ cells is around 85%; although not all STEP cells also express CRHR1. These results indicate STEP indeed acts through CRHR1 to suppress CRH signaling in the ovBNST.

**10. CRHR1 co-localizes with CRH in the mice BNST:**

As shown in Fig.S10C and 10F, most CRH cells (red; Fig.S10A and 10D) co-localize with CRHR1 (green; Fig.S10B and 10E) (around 75-85% co-localization) in the mice BNST. These results indicate CRH acted via CRHR1 in the BNST to exert its functions.

**Figure Legends:**

**Fig S1**: **Comparison of body weight at PND70, total distance mice traveled in the open filed (OF) test and total entry frequency in the elevated plus maze (EPM) test between Control vs. maternal separated (MS) group of mice.**

(A): Comparison of body weight at postnatal day 70 (PND70) reveals no significant difference between Control group and maternal deprived (MS) group.

(B): Comparison of total distance that mice traveled in the OF test revealed no significant difference between Control vs. MS group;

(C): No significant difference in the total entry frequency that mice traveled in the elevated plus maze (EPM) test was found between Control vs. MS group.

NS: no significant difference (p>0.05).

**Fig S2:** **Comparison of the dam’s maternal care behaviors towards the pups between MS vs. Control group of mice from postnatal day 4 to 11 (PND4-11).**

(A): Maternal licking and grooming behavior compared between MS (n=10 litters) vs. Control (n=10 litters) mice;

(B): Total nursing time of the dam compared between MS (n=10 litters) vs. Control (n=10 litters) mice;

(C): The time that the dam is leaving off her pups compared between MS (n=10 litters) vs. Control (n=10 litters) mice.

No significant difference (p>0.05) was found between MS vs. Control dam for all the parameters examined.

**Fig S3**: **Comparison of typical immunostaining example for** **c-fos (A and B), CRH (C and D), PACAP (E and F) and STEP (G and H) in the antero-dorsolateral BNST (BNSTadl) of Control vs. MS mice.**

(A) and (B): c-fos-immunostaining pattern compared in the BNSTadl of (A) Control (left) vs. (B) MD (right) mice;

(C) and (D): CRH-immunostaining pattern compared in the BNSTadl of (C) Control (left) vs. (D) MD (right) mice;

(E) and (F): PACAP-immunostaining pattern compared in the BNSTadl of (E) Control (left) vs. (F) MD (right) mice;

(G) and (H): STEP-immunostaining pattern compared in the BNSTadl of (G) Control (left) vs. (H) MD (right) mice.

scale bar 100 μm; ic: internal capsule; ac: anterior commissure. White arrows and arrowheads point to typical c-fos-, CRH-, PACAP- and STEP-immunopositive cells in the BNSTadl.

**Fig S4**: **Comparison of c-fos- (A), CRH- (B), PACAP- (C) and STEP (D)-immunoreactive (IR) cell number in the total anterior BNST and adBNST (antero-dorsal region closely surrounding ovBNST) of Control vs. MS group.**

(A): Significant higher CRH^+^ cell number in the total anterior BNST was found in the MS group vs. Control group (p<0.01);

(B): Significant higher PACAP^+^ cell number in the total anterior BNST was found in the MS group vs. Control group (p<0.001);

(C): Significant higher STEP^+^ cell number in the total anterior BNST was found in the MS group vs. Control group (p<0.001);

(D): Anatomy example of the anterolateral dorsal region of BNST (adBNST), the region closely surrounding oval nucleus in the BNSTadl.

(E): No significant difference was found in the c-fos^+^ cells number in the adBNST between Control vs. MS group (p>0.05) ;

(F): No significant difference was found in the CRH^+^ cell number in the adBNST between Control vs. MS group (p>0.05) ;

(G): No significant difference was found in the PACAP^+^ cell number in the adBNST between Control vs. MS group (p>0.05);

(H): No significant difference was found in the STEP^+^ cell number in the adBNST between Control vs. MS group (p>0.05).

ic: internal capsule; ac: anterior commissure; ov: oval nucleus; ad: anterolateral dorsal; n=6 mice for each MS and Control group; *: p<0.05; **: p<0.01; ***: p<0.001; NS: non-significant different.

**Fig S5**: Total GluR1 protein expression level has no significant difference in the BNST of Control (n=7 mice) vs. MS (n=7 mice) group of mice.

NS: non-significant different (p>0.05).

**Fig S6**: **Maladaptive behavioral effects of MS persisted when** **CRHR1 was blocked by R121919 but PKA was simultaneously activated by forskolin**.

(A): No significant difference was found in the EPM open arm duration between MS group vs. MS+R121919+forskolin group.

(B): No significant difference was found in the EPM open arm entry frequency between MS group vs. MS+R121919+forskolin group.

(C): No significant difference was found in the OF center duration between MS group vs. MS+R121919+forskolin group.

(D): No significant difference was found in the OF center distance between MS group vs. MS+R121919+forskolin group.

(E): No significant difference was found in the OF center entry frequency between MS group vs. MS+R121919+forskolin group.

(F): No significant difference was found in the NSF latency time between MS group vs. MS+R121919+forskolin group.

(G): No significant difference was found in the sucrose preference between MS group vs. MS+R121919+forskolin group.

n=10 for MS group; n=7 for MS+R121919+forskolin group; NS: non-significant different.

**Fig S7**: **When CRHR1 was blocked by R121919 in the MS mice, the maladaptive behavioral effect of co-infused BDNF was abolished by co-infusion of PKC-selective antagonist GF109203X into ovBNST of MS mice**.

(A): Significantly increased open arm duration was found in the MS+R121919+BDNF+GF109203X group (n=7) compared with MS+R121919+BDNF group (n=7) in the EPM test ;

(B): Again, significantly increased open arm entry frequency was found in the MS+R121919+BDNF+GF109203X group (n=7) compared with MS+R121919+BDNF group (n=7) in the EPM test;

(C): A significant increase in the center duration was found in the MS+R121919+BDNF+GF109203X group (n=7) compared with MS+R121919+BDNF group (n=7) in the OF test;

(D): Significantly increased center travel distance was found in the MS+R121919+BDNF+GF109203X group (n=7) compared with MS+R121919+BDNF group (n=7) in the OF test;

(E): Significantly increased center entry frequency was found in the MS+R121919+BDNF+GF109203X group (n=7) compared with MS+R121919+BDNF group (n=7) in the OF test;

(F): A significant decrease in the latency time to eat was found in the MS+R121919+BDNF+GF109203X group (n=7) compared with MS+R121919+BDNF group (n=7) in the NSF test;

(G): Again, a significantly increase in the sucrose preference percentage was found in the MS+R121919+BDNF+GF109203X group (n=7) compared with MS+R121919+BDNF group (n=7) in the sucrose preference test.

n=7 for both MS+R121919+BDNF and MS+R121919+BDNF+GF109203X group; NS: non-significant different; *: p<0.05; **: p<0.01; ***: p<0.001.

**Fig S8**: **Example of co-immunofluorescence staining of CRH (A and D, red) and PACAP (B, green), and STEP (E, green) signaling in the oval nucleus of BNST (ovBNST) shows co-cololization of CRH and PACAP (C) and CRH and STEP (F) in the ovBNST.**

Scale bar: 50 μm.

**Fig S9:** **Immunofluorescence figure shows co-localization (C; yellow) of CRHR1 (A; green) with STEP (B; red) in the ovBNST:**

Scale bar: 50 μm.

**Fig S10**: **Immunofluorescence figure shows most CRHR1-immunopositive cells (green; B and E) co-localize (yellow; C and E) with CRH-immunopositive cells (red; A and D) in the mice BNST.**

Scale bar: A-C: 100 μm; D-F: 50 μm.

**References:**

1. Samuels, B.A.*, et al.* 5-HT1A receptors on mature dentate gyrus granule cells are critical for the antidepressant response. *Nat Neurosci* **18**, 1606-1616 (2015).

2. Samuels, B.A.*, et al.* The Behavioral Effects of the Antidepressant Tianeptine Require the Mu-Opioid Receptor. *Neuropsychopharmacology* **42**, 2052-2063 (2017).

3. Lesuis, S.L., van Hoek, B., Lucassen, P.J. & Krugers, H.J. Early postnatal handling reduces hippocampal amyloid plaque formation and enhances cognitive performance in APPswe/PS1dE9 mice at middle age. *Neurobiol Learn Mem* **144**, 27-35 (2017).

4. Livak, K.J. & Schmittgen, T.D. Analysis of relative gene expression data using real-time quantitative PCR and the 2(-Delta Delta C(T)) Method. *Methods* **25**, 402-408 (2001).

5. Hu, P.*, et al.* Chronic Stress Induces Maladaptive Behaviors by Activating Corticotropin-Releasing Hormone Signaling in the Mouse Oval Bed Nucleus of the Stria Terminalis. *J Neurosci* **40**, 2519-2537 (2020).

6. Hu, P.*, et al.* Gq Protein-Coupled Membrane-Initiated Estrogen Signaling Rapidly Excites Corticotropin-Releasing Hormone Neurons in the Hypothalamic Paraventricular Nucleus in Female Mice. *Endocrinology* **157**, 3604-3620 (2016).

7. Roepke, T.A., Qiu, J., Smith, A.W., Ronnekleiv, O.K. & Kelly, M.J. Fasting and 17beta-estradiol differentially modulate the M-current in neuropeptide Y neurons. *J Neurosci* **31**, 11825-11835 (2011).

8. Pomrenze, M.B.*, et al.* A Corticotropin Releasing Factor Network in the Extended Amygdala for Anxiety. *J Neurosci* **39**, 1030-1043 (2019).

9. Saylor, A.J. & McGinty, J.F. An intrastriatal brain-derived neurotrophic factor infusion restores striatal gene expression in Bdnf heterozygous mice. *Brain Struct Funct* **215**, 97-104 (2010).

10. Sun, W.L., Eisenstein, S.A., Zelek-Molik, A. & McGinty, J.F. A single brain-derived neurotrophic factor infusion into the dorsomedial prefrontal cortex attenuates cocaine self-administration-induced phosphorylation of synapsin in the nucleus accumbens during early withdrawal. *Int J Neuropsychopharmacol* **18**(2014).

11. Gabriel, L.R., Wu, S. & Melikian, H.E. Brain slice biotinylation: an ex vivo approach to measure region-specific plasma membrane protein trafficking in adult neurons. *J Vis Exp* (2014).

12. Dabrowska, J.*, et al.* Striatal-enriched protein tyrosine phosphatase-STEPs toward understanding chronic stress-induced activation of corticotrophin releasing factor neurons in the rat bed nucleus of the stria terminalis. *Biol Psychiatry* **74**, 817-826 (2013).

13. Hammack, S.E.*, et al.* Roles for pituitary adenylate cyclase-activating peptide (PACAP) expression and signaling in the bed nucleus of the stria terminalis (BNST) in mediating the behavioral consequences of chronic stress. *J Mol Neurosci* **42**, 327-340 (2010).

14. Roman, C.W.*, et al.* PAC1 receptor antagonism in the bed nucleus of the stria terminalis (BNST) attenuates the endocrine and behavioral consequences of chronic stress. *Psychoneuroendocrinology* **47**, 151-165 (2014).

15. Stemmelin, J., Mathis, C. & Ungerer, A. GF 109203X, a selective inhibitor of protein kinase C, impairs retention performance in an operant task. *Neuroreport* **10**, 2805-2809 (1999).
