# Supplementary material for: Early-life stress alters affective behaviors in adult mice through persistent activation of CRH-BDNF signaling in the oval bed nucleus of the stria terminalis
Source: Transl Psychiatry. 2020 Nov 11;10:396. doi: 10.1038/s41398-020-01070-3 (PMC7658214; doi:10.1038/s41398-020-01070-3)
Supplement: Supplementary file 2 — Supplemental Figure 1 [file 41398_2020_1070_MOESM2_ESM.pdf]

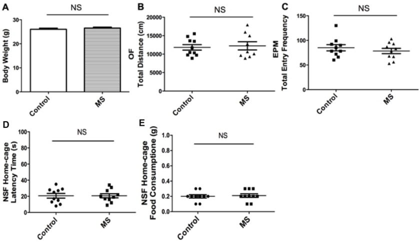

A
NS
Body Weight (g)
30
20
10
0
Control
MS
B
NS
OF
Total Distance (cm)
20000
15000
10000
5000
0
Control
MS
C
NS
EPM
Total Entry Frequency
150
100
50
0
Control
MS
D
NS
NSF Home-cage
Latency Time (s)
100
80
60
40
20
0
Control
MS
E
NS
NSF Home-cage
Food Consumptione (g)
1.0
0.8
0.6
0.4
0.2
0.0
Control
MS
