# Supplementary figures and images for: Early-life stress alters affective behaviors in adult mice through persistent activation of CRH-BDNF signaling in the oval bed nucleus of the stria terminalis
Source: Transl Psychiatry. 2020 Nov 11;10:396. doi: 10.1038/s41398-020-01070-3 (PMC7658214; doi:10.1038/s41398-020-01070-3)

**A****Licking and Grooming**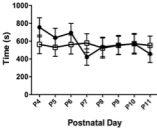**B****Nursing**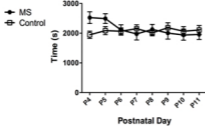**C****Time Off Pups**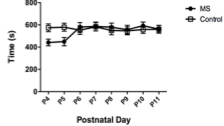

Supplement: Supplementary file 3 — Supplemental Figure 2 [file 41398_2020_1070_MOESM3_ESM.pdf]

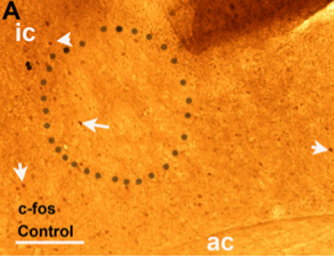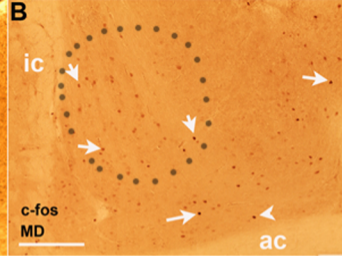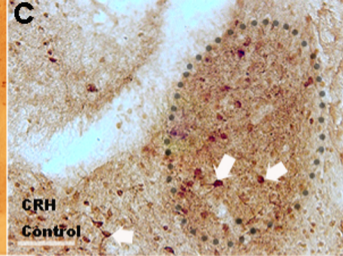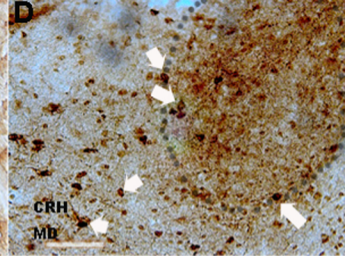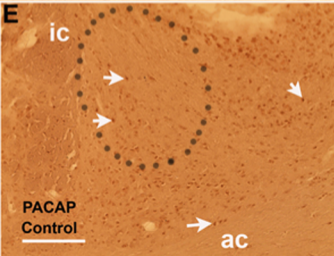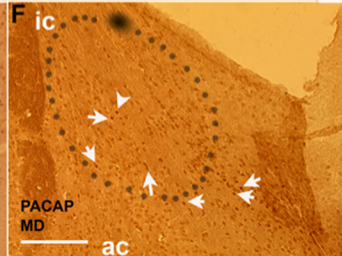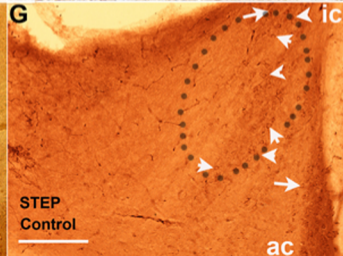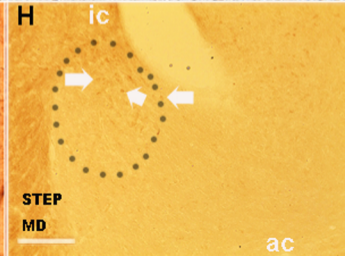

Supplement: Supplementary file 4 — Supplemental Figure 3 [file 41398_2020_1070_MOESM4_ESM.pdf]

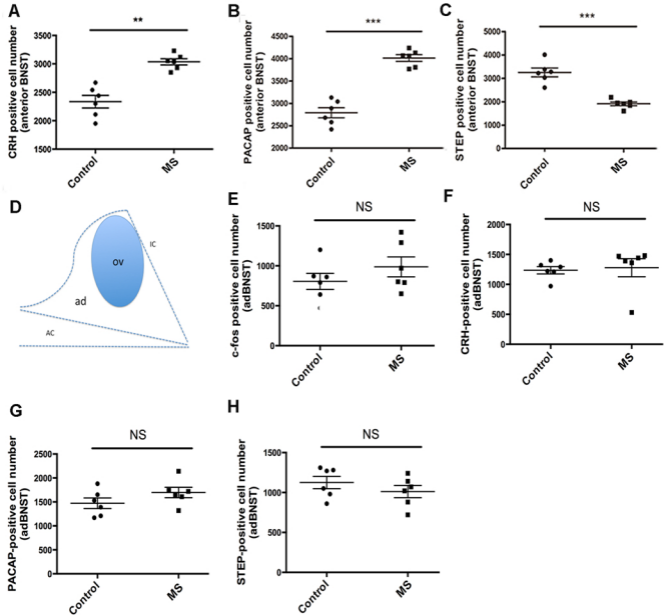

Supplement: Supplementary file 5 — Supplemental Figure 4 [file 41398_2020_1070_MOESM5_ESM.pdf]

**Total GluR1 expression level  
(%GAPDH)**

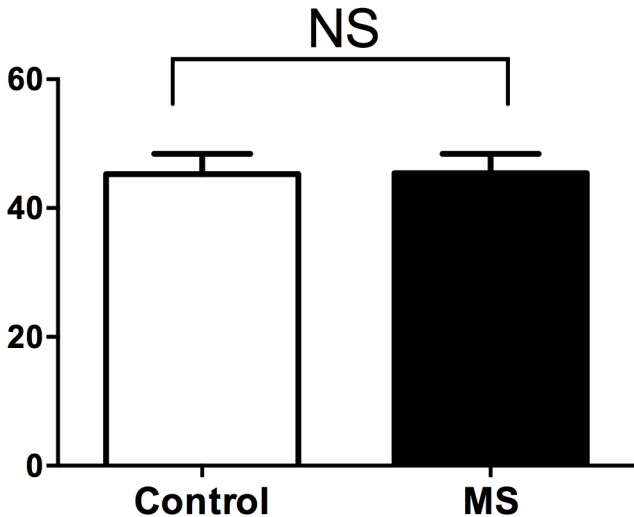

Supplement: Supplementary file 6 — Supplemental Figure 5 [file 41398_2020_1070_MOESM6_ESM.pdf]

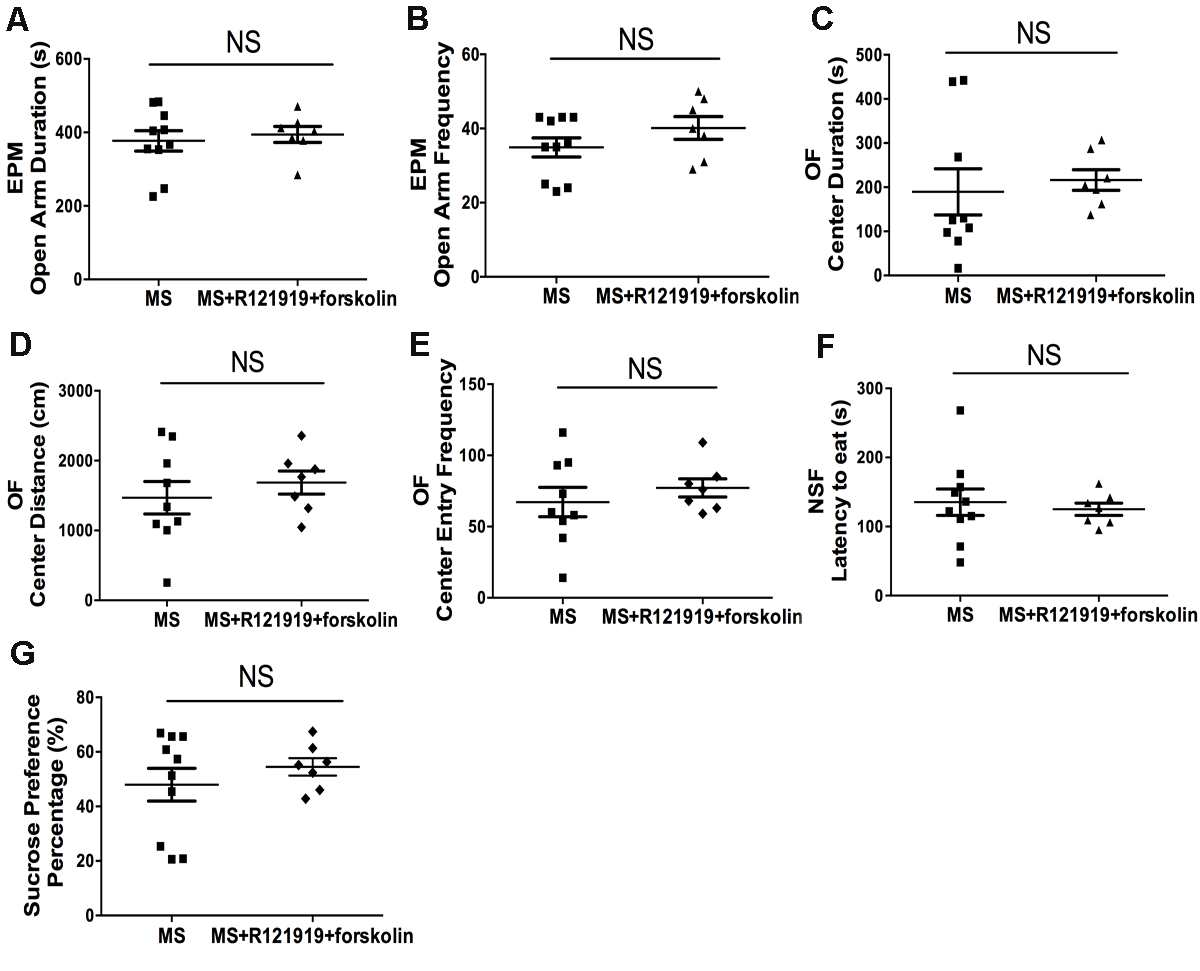

Supplement: Supplementary file 7 — Supplemental Figure 6 [file 41398_2020_1070_MOESM7_ESM.tif]

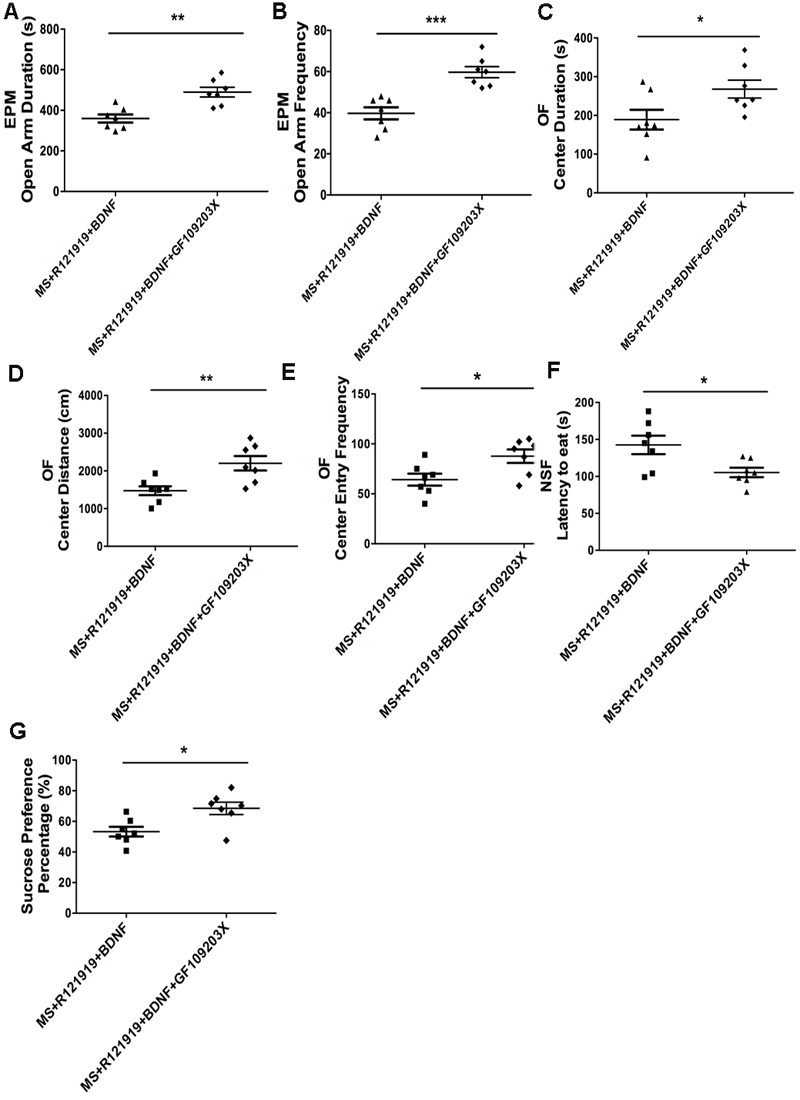

Supplement: Supplementary file 8 — Supplemental Figure 7 [file 41398_2020_1070_MOESM8_ESM.tif]

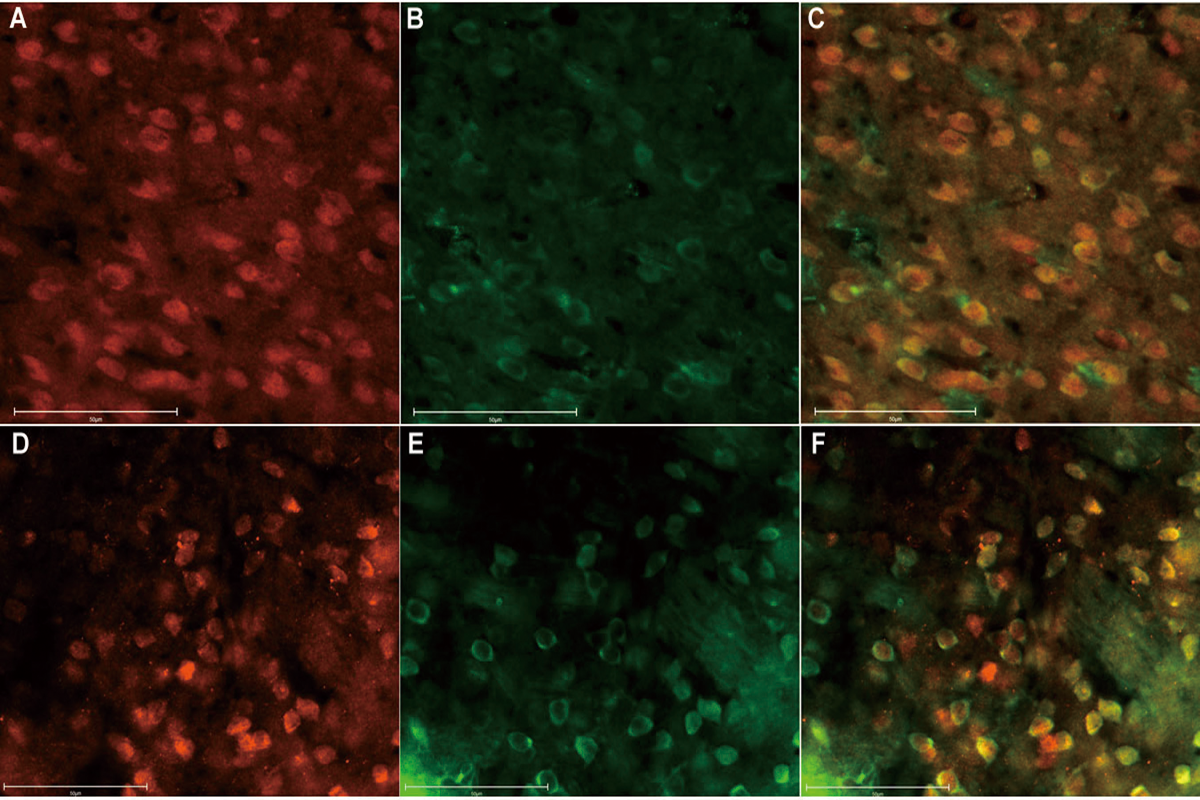

Supplement: Supplementary file 9 — Supplemental Figure 8 [file 41398_2020_1070_MOESM9_ESM.tif]

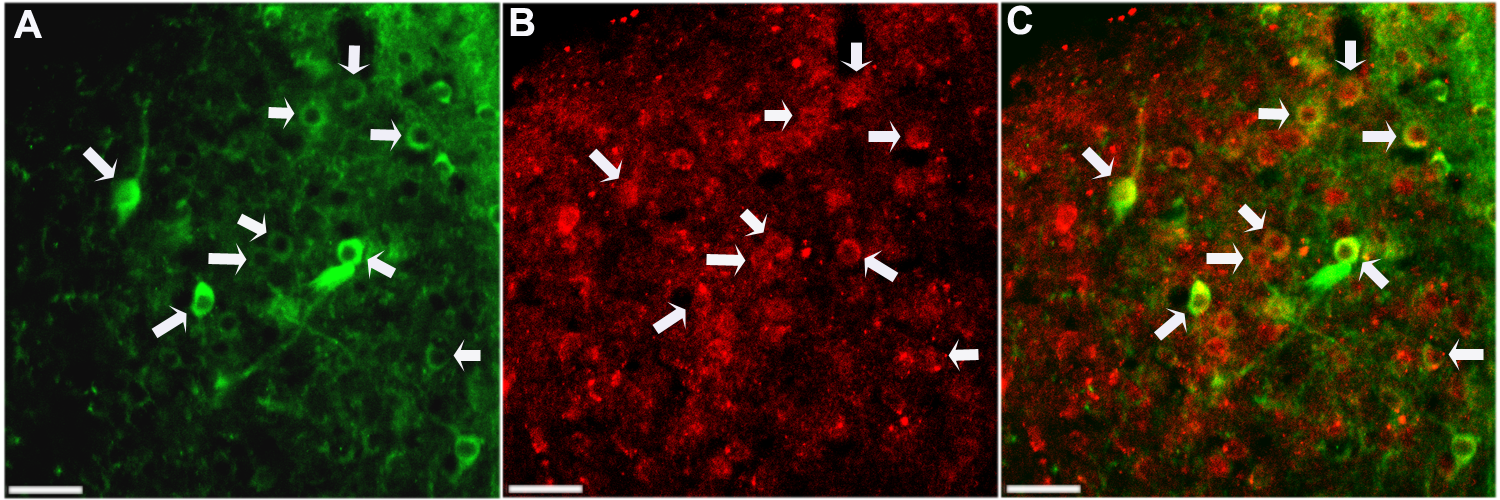

Supplement: Supplementary file 10 — Supplemental Figure 9 [file 41398_2020_1070_MOESM10_ESM.tif]

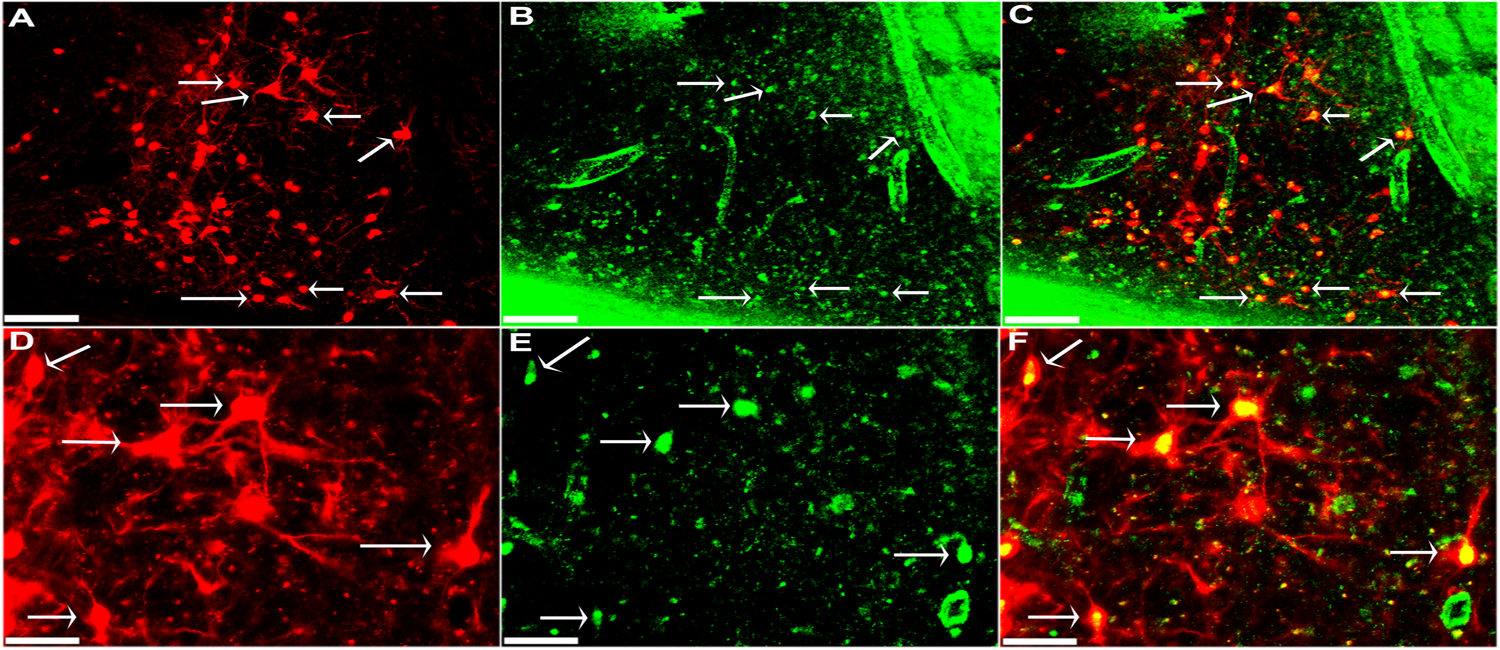

Supplement: Supplementary file 11 — Supplemental Figure 10 [file 41398_2020_1070_MOESM11_ESM.tif]
